# Neoadjuvant nivolumab and chemotherapy in early estrogen receptor-positive breast cancer: a randomized phase 3 trial

**DOI:** 10.1038/s41591-024-03414-8

**Published:** 2025-01-21

**Authors:** Sherene Loi, Roberto Salgado, Giuseppe Curigliano, Roberto Iván Romero Díaz, Suzette Delaloge, Carlos Ignacio Rojas García, Marleen Kok, Cristina Saura, Nadia Harbeck, Elizabeth A. Mittendorf, Denise A. Yardley, Alberto Suárez Zaizar, Facundo Rufino Caminos, Andrei Ungureanu, Joaquin G. Reinoso-Toledo, Valentina Guarneri, Daniel Egle, Felipe Ades, Misena Pacius, Aparna Chhibber, Rajalakshmi Chandra, Raheel Nathani, Thomas Spires, Jenny Qun Wu, Lajos Pusztai, Heather McArthur

**Affiliations:** 1 Peter MacCallum Cancer Centre, Melbourne, Victoria Australia; 2 University of Melbourne, Parkville, Victoria Australia; 3 Department of Pathology, ZAS Hospitals, Antwerp, Belgium; 4 European Institute of Oncology, IRCCS, Milan, Italy; 5 University of Milan, Milan, Italy; 6 Consultorio de Oncólogo Médico, Oaxaca, Mexico; 7 Gustave Roussy Cancer Campus, Villejuif, France; 8 Bradford Hill Investigación Clinica, Región Metropolitana, Santiago, Chile; 9 Netherlands Cancer Institute, Amsterdam, the Netherlands; 10 Vall d’Hebron University Hospital, Vall d’Hebron Institute of Oncology (VHIO), Barcelona, Spain; 11 Breast Center, Department of Obstetrics and Gynecology and CCC Munich, Ludwig Maximilians University Hospital, Munich, Germany; 12 Dana-Farber Brigham Cancer Center, Boston, MA USA; 13 Sarah Cannon Research Institute, Nashville, TN USA; 14 CENEIT Oncológicos, Mexico City, Mexico; 15 Hospital Italiano De Córdoba, Córdoba, Argentina; 16 Radiotherapy Center CLUJ S.R.L., Florești, Romania; 17 Consultorio del Dr. Joaquin Gabriel Reinoso Toledo, Monterrey, Mexico; 18 Istituto Oncologico Veneto IRCCS, Padua, Italy; 19 Department of Surgery, Oncology and Gastroenterology, University of Padova, Padova, Italy; 20 Department of Gynecology, Medical University of Innsbruck, Innsbruck, Austria; 21 ABCSG – Austrian Breast & Colorectal Cancer Study Group, Vienna, Austria; 22 Bristol Myers Squibb, Princeton, NJ USA; 23 Smilow Cancer Hospital at Yale, New Haven, CT USA; 24 University of Texas Southwestern Medical Center, Dallas, TX USA

**Keywords:** Medical research, Cancer

## Abstract

Patients with estrogen receptor-positive (ER+), human epidermal growth factor receptor 2-negative (HER2−) primary breast cancer (BC) have low pathological complete response (pCR) rates with neoadjuvant chemotherapy. A subset of ER+/HER2− BC contains dense lymphocytic infiltration. We hypothesized that addition of an anti-programmed death 1 agent may increase pCR rates in this BC subtype. We conducted a randomized, multicenter, double-blind phase 3 trial to investigate the benefit of adding nivolumab to neoadjuvant chemotherapy in patients with newly diagnosed, high-risk, grade 3 or 2 (ER 1 to ≤10%) ER+/HER2− primary BC. In total, 510 patients were randomized to receive anthracycline and taxane-based chemotherapy with either intravenous nivolumab or placebo. The primary endpoint of pCR was significantly higher in the nivolumab arm compared with placebo (24.5% versus 13.8%; *P* = 0.0021), with greater benefit observed in patients with programmed death ligand 1-positive tumors (VENTANA SP142 ≥1%: 44.3% versus 20.2% respectively). There were no new safety signals identified. Of the five deaths that occurred in the nivolumab arm, two were related to study drug toxicity; no deaths occurred in the placebo arm. Adding nivolumab to neoadjuvant chemotherapy significantly increased pCR rates in high-risk, early-stage ER+/HER2− BC, particularly among patients with higher stromal tumor-infiltrating lymphocyte levels or programmed death ligand 1 expression, suggesting a new treatment paradigm that emphasizes the role of immunotherapy and T cell immunosurveillance in luminal disease. Clinical trials.gov identifier: NCT04109066

## Abstract (web summary)

In the CheckMate 7FL trial, neoadjuvant nivolumab and chemotherapy in patients with newly diagnosed, high-risk estrogen receptor-positive breast cancer led to an increased pathological complete response rate compared with neoadjuvant chemotherapy alone, and this increase was associated with immune-related biomarkers and estrogen receptor expression.

## Main

Approximately 2.3 million cases of BC were diagnosed globally in 2020, of which 70% were the ER+/HER2− subtype^1,2^. ER+/HER2− BC exhibits significant heterogeneity in its responses to treatment and clinical outcomes, posing substantial challenges for effective management. This heterogeneity may be caused by distinct differences in the molecular subtypes of ER+/HER2− BC, including subtypes with varying estrogen and progesterone receptor expression, and those that are immunogenic, proliferative and receptor tyrosine kinase-driven, which require specific treatments^3^.

Current systemic therapeutic strategies for high-risk, early-stage ER+/HER2− BC include: neoadjuvant or adjuvant chemotherapy (CT); prolonged adjuvant endocrine therapy (ET) with or without adjuvant targeted therapies, including cyclin-dependent kinase 4/6 inhibitors; and poly(ADP-ribose) polymerase inhibitors for patients carrying germline pathogenic *BRCA* alterations^4–8^.

Anti-programmed death ligand 1 (PD-L1) agents significantly improve clinical outcomes in early-stage triple-negative BC (TNBC) and PD-L1+ metastatic TNBC^9–11^. A subset of ER+/HER2− BC contains a dense lymphocytic infiltration, similar to that seen in TNBC^12,13^; however, it is unclear how this relates to the response to immune checkpoint inhibitors in ER+/HER2− BC^14–16^. Results from the adaptively randomized I-SPY2 study suggest that anti-PD-(L)1 agents have the potential to increase the proportion of patients with high-risk ER+/HER2− BC who achieve pCR or minimal residual disease (residual cancer burden (RCB) score of 0 or I) following neoadjuvant treatment^10,17^. The CheckMate 7FL (NCT04109066) study aimed to investigate the benefit of adding nivolumab to neoadjuvant CT followed by adjuvant ET in patients with newly diagnosed early-stage high-risk ER+/HER2− BC. We also sought to define patient subpopulations most likely to respond to nivolumab in combination with neoadjuvant CT.

## Results

### Study population and demographics

**Fig. 1:**
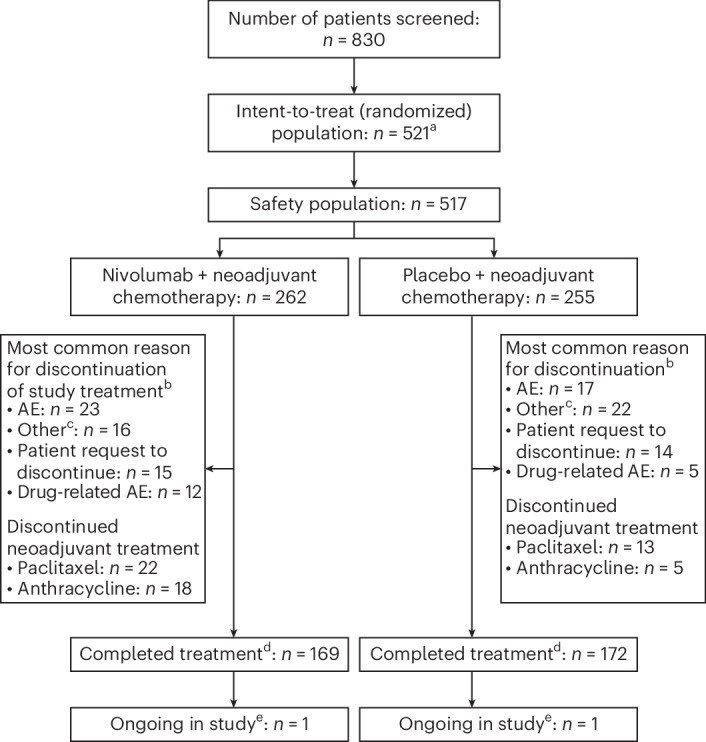
Flow chart showing patient disposition. Twenty-five patients received abemaciclib, and may have received it after neoadjuvant treatment or discontinued adjuvant treatment to receive abemaciclib. ^a^The mITT population comprised 510 patients (257 patients in the nivolumab arm and 253 patients in the placebo arm). Because of the sponsor’s decision to close Russian sites, 11 patients were excluded owing to insufficient follow-up for pCR. ^b^Discontinuation of study treatment included treatment discontinuation during the adjuvant phase. ^c^Most common reasons for discontinuation of treatment captured by ‘Other’ were disease progression, principal investigator discretion, serious AEs or AEs and withdrawal of consent. ^d^Completers were patients who completed surgery and the adjuvant phase. ^e^Patients were reported as ongoing at the time of the premature closure of Russian sites.

From 20 November 2019 to 7 April 2022, 830 patients were screened at 221 sites in 31 countries. Of the 830 patients screened, 521 were randomized. Because of the sponsor’s decision to close all sites in Russia after the Ukraine–Russia geopolitical conflict began, 11 patients were excluded from the analysis population because of insufficient follow-up for pCR assessment. The resulting population formed the modified intent-to-treat population (mITT), which comprised 510 patients who received neoadjuvant CT with either nivolumab (*n* = 257) or placebo (*n* = 253). The safety population consisted of the 517 patients who received neoadjuvant CT with either nivolumab (*n* = 262) or placebo (*n* = 255) (Fig. 1). Patient demographic and clinical characteristics were balanced between the two treatment arms (Table 1).

**Table 1:** Baseline patient demographics and clinical characteristics (mITT population)

Demographic/characteristic	Nivolumab plus neoadjuvant CT			Placebo plus neoadjuvant CT		
	mITT population (*n* = 257)	SP142 PD-L1− (*n* = 169)^a^	SP142 PD-L1+ (*n* = 88)^a^	mITT population (*n* = 253)	SP142 PD-L1− (*n* = 169)^a^	SP142 PD-L1+ (*n* = 84)^a^
Female	257 (100)	169 (100)	88 (100)	252 (99.6)	168 (99.4)	84 (100)
Median age, years (range)	50 (24–78)	51 (24–77)	49 (28–78)	51 (23–79)	51 (23–79)	51 (27–78)
ECOG PS						
0	221 (86)	144 (85)	77 (88)	222 (88)	146 (86)	76 (91)
1	36 (14)	25 (15)	11 (13)	31 (12)	23 (14)	8 (10)
Tumor grade^**b**^						
Grade 2	6 (2)	2 (1)	4 (5)	1 (<1)	1 (1)	0 (0)
Grade 3	251 (98)	167 (99)	84 (96)	252 (>99)	168 (99)	84 (100)
Stage^**c**^ (cTNM classification^**d**^)						
Stage II	135 (53)	88 (52)	47 (53)	138 (55)	94 (56)	44 (52)
Stage III	118 (46)	77 (46)	41 (47)	105 (42)	67 (40)	38 (45)
Not assigned/reported	4 (2)	4 (2)	0 (0)	7 (3)	6 (4)	1 (1)
PD-L1^**b**^						
<1%	169 (66)	–	–	169 (67)	–	–
≥1%	88 (34)			84 (33)		
Axillary nodal status						
Positive	205 (80)	135 (80)	70 (80)	201 (79)	134 (79)	67 (80)
Negative	52 (20)	34 (20)	18 (21)	52 (21)	35 (21)	17 (20)
AC dose-frequency CT regimen^e^						
Q2W	132 (51)	85 (50)	47 (53)	134 (53)	88 (52)	46 (55)
Q3W	125 (49)	84 (50)	41 (47)	119 (47)	81 (48)	38 (45)

All values are given as *n* (%), unless stated otherwise.

^a^PD-L1–expressing tumor-infiltrating IC as percentage of tumor area (PD-L1− defined as PD-L1 IC <1%; PD-L1+ defined as PD-L1 IC ≥1%) using the VENTANA SP142 assay, per central assessment.

^b^Locally assessed.

^c^Arm B included one patient with stage I disease and two patients with stage IV disease, who were deemed eligible and later recategorized as having stage II disease.

^d^American Joint Committee on Cancer Cancer Staging Manual, 8th edition.

^e^Gonadotropin-releasing hormone agonist therapy was allowed for ovarian preservation.

AC, anthracycline + cyclophosphamide; cTNM, clinical TNM staging system (T size and extent of primary tumor; N extent of spread to the lymph nodes; M presence of metastasis); ECOG PS, Eastern Cooperative Oncology Group performance status; QXW, every X weeks.

In the safety population, the mean (min, max) treatment duration during the paclitaxel neoadjuvant phase was 11.0 (1.1, 16.1) weeks for patients receiving nivolumab and 11.2 (1.0, 15.6) weeks for patients receiving placebo. The mean (min, max) treatment duration during the anthracycline neoadjuvant phase was 7.6 (0.1, 13.0) weeks for patients receiving nivolumab and 7.7 (0.1, 15.1) weeks for patients receiving placebo. Of patients randomized to nivolumab and placebo, respectively, 89% (233 of 263) and 91% (236 of 258) underwent surgery (Supplementary Table 1).

### Efficacy

**Fig. 2:**
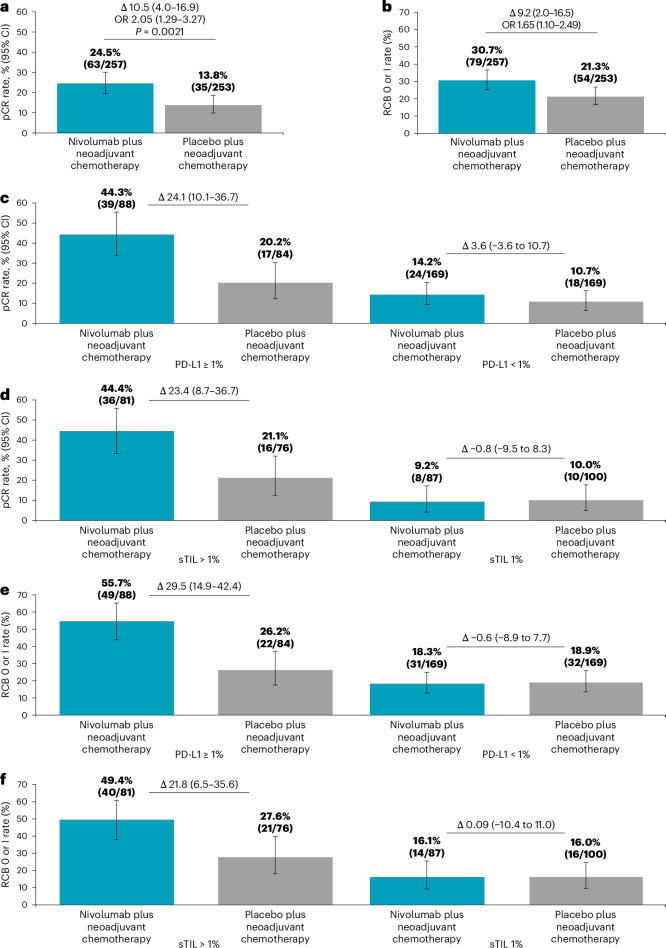
Efficacy endpoints for the overall population and by subgroups. **a**,**b**, Proportion of patients with pCR (**a**) and RCB 0 or I (**b**) for the nivolumab plus neoadjuvant CT (*N* = 257) and placebo plus neoadjuvant CT (*N* = 253) arms in the mITT population. **c**,**d**, Proportion of patients with pCR in the nivolumab plus neoadjuvant CT and placebo plus neoadjuvant CT arms by PD-L1 status ≥1% (*n* = 88, *n* = 84) or <1% (*n* = 169, *n* = 169) (**c**) and stromal tumor infiltrating lymphocyte (sTIL) status >1% (*n* = 81, *n* = 76) or ≤1% (*n* = 87, *n* = 100) (**d**). **e**,**f**, Proportion of patients with RCB 0 or I rate by PD-L1 status ≥1% (*n* = 88, *n* = 84) or <1% (*n* = 169, *n* = 169) (**e**) and sTIL status >1% (*n* = 81, *n* = 76) or ≤1% (*n* = 87, *n* = 100) (**f**). Data are presented as percentages with error bars showing the 95% CI around the observed proportion of patients in the treatment arm. The CIs for each treatment arm were calculated using the Clopper–Pearson method and CIs for differences (Δ) between treatment arms were calculated using the Newcombe method without continuity correction. Strata-adjusted difference in pCR rate between the two arms was analyzed with the stratified Cochran–Mantel–Haenszel method of weighting with a two-sided alpha level of 0.05 (**a**). Strata-adjusted OR was assessed with the Mantel–Haenszel method (**a**,**b**). The number of patients with pCR or RCB 0 or I (*n*) and the total number of patients in each subgroup (*N*) are shown above each bar. Database lock: 14 April 2023 (**a**,**b**) and 20 March 2024 (**d**–**f**). *n*, number of patients with pCR or RCB 0/I; *N*, number of patients in each treatment group.

A statistically significantly higher proportion of patients who received nivolumab achieved pCR (ypT0/is, ypN0; 24.5%, 63 of 257) versus placebo (13.8%, 35 of 253; odds ratio (OR) 2.05 (95% confidence interval (CI) 1.29 to 3.27, *P* = 0.0021) in addition to neoadjuvant CT (Fig. 2a).

**Fig. 3:**
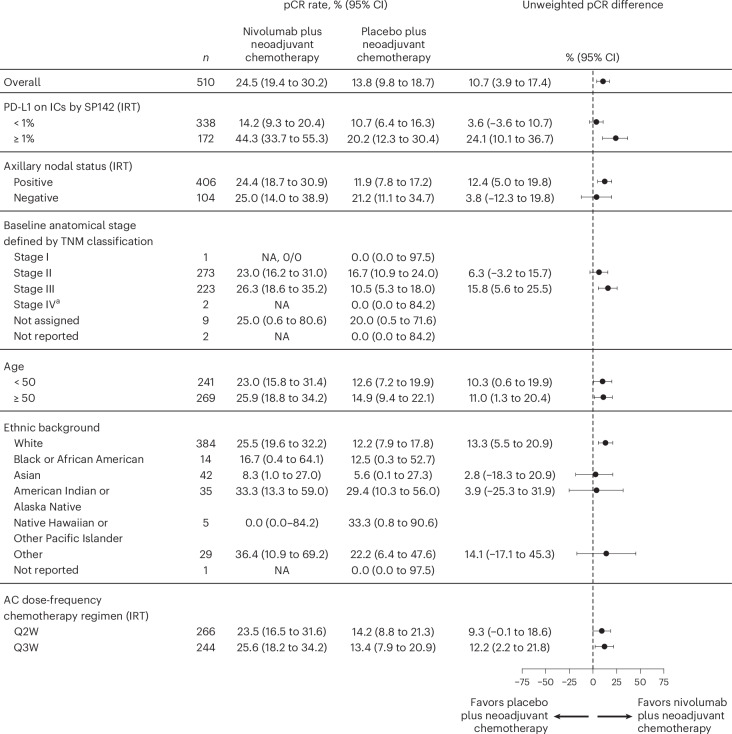
Forest plot of proportion of patients with pCR in the nivolumab plus neoadjuvant CT and placebo plus neoadjuvant CT arms by subgroup analyses. Data are presented as percentages with error bars showing the 95% CIs around the observed proportion of patients in the treatment arm. The CIs for each treatment arm were calculated using the Clopper–Pearson method and CIs for differences between treatment arms were calculated using the Newcombe method without continuity correction. pCR rate difference was not computed for subsets with fewer than 10 patients per treatment arms. ^a^The two patients who were initially categorized as having stage IV disease were deemed eligible and later recategorized as having stage II disease. AC, anthracycline; IRT, interactive response technology; *n*, total number of patients in subgroup; NA, not available; Q2W, every 2 weeks; Q3W every 3 weeks. Database lock: 14 April 2023.

The proportion of patients who experienced pCR was numerically higher among those who had PD-L1+ tumors (PD-L1-expressing tumor-infiltrating immune cells (IC) ≥ 1% IC, *n* = 172) versus those with PD-L1− tumors (<1% IC, *n* = 338). The difference in pCR rates (95% CI) between the nivolumab arm and placebo arm was 24.1% (10.1 to 36.7) and 3.6% (−3.6 to 10.7) for PD-L1+ and PD-L1− tumors, respectively (Fig. 2c). Subgroup analyses of pCR rates were consistent with these results (Fig. 3).

RCB 0 or I rates in the mITT population and by PD-L1 status, as well as subgroup analyses, were consistent with the findings observed for pCR (Fig. 2b,e and Supplementary Fig. 1). Nivolumab skewed the distribution of RCB toward the lower classes versus placebo (Supplementary Fig. 2).

Because of its early termination, the study was significantly underpowered for event-free survival (EFS), and median follow-up for EFS in the mITT population at reporting was premature at 19 months, with a low number of events observed. Results of a descriptive exploratory analysis showed that EFS was similar between the two treatment arms, with an 18-month rate of 89.1% (95% CI 83.8 to 92.7) in the nivolumab arm and 91.7% (95% CI 86.7 to 94.8) in the placebo arm (Supplementary Fig. 3).

### Efficacy according to immune biomarkers

**Fig. 4:**
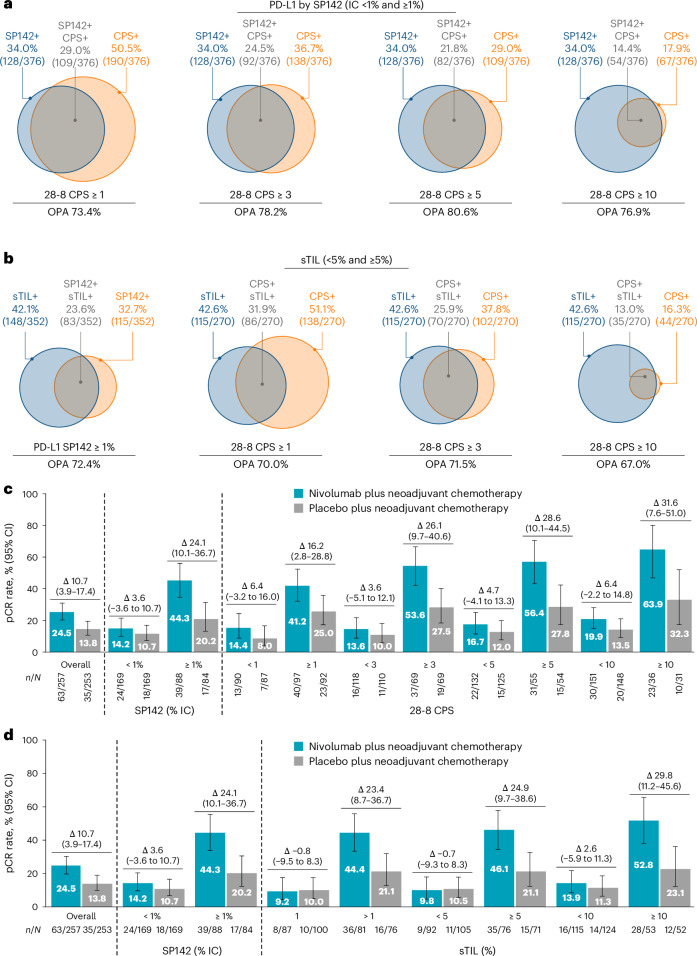
Efficacy of nivolumab by subgroups. **a**, Concordance between PD-L1 assays SP142 and 28-8 CPS. **b**, sTIL and PD-L1 expression in patients with quantifiable sTIL and PD-L1 by SP142 or 28-8 CPS. **c**, pCR rates in the nivolumab plus neoadjuvant CT and placebo plus neoadjuvant CT arms by PD-L1 status as determined by the SP142 (IC%) and 28-8 CPS (cutoffs 1–20) assays. **d**, PD-L1 status as determined by the SP142 (IC%) assay and percentage of sTIL (cutoffs 1%, 5%, 10%). The number of patients with qualifying data (*n*) and the total number of patients in each subgroup (*N*) are shown above each circle (**a**,**b**) or below each bar (**c**,**d**). Data are presented as percentages. CIs for the observed proportion of patients in the treatment arm were calculated using the Clopper–Pearson method and CIs for differences between treatment arms were calculated using the Newcombe method without continuity correction (**c**,**d**). Vertical dashed lines are to visually distinguish between Overall, SP142 and 28-8 in **c**, and Overall, SP142 and sTIL in **d**. 28-8 CPS, Dako 28-8 assay using the CPS algorithm; OPA, overall percentage agreement; SP142 VENTANA, PD-L1 SP142 assay. Clinical database; SP142 cutoff at ≥1% versus <1%. Because of the small sample size, the percent agreement was not calculated for CPS ≥ 20). Database lock: 20 March 2024. Additional patients were included in the CPS-evaluable group at this final database lock.

The prevalence of the PD-L1+ population in the two arms was balanced, as evaluated by baseline PD-L1 expression status, defined by either VENTANA SP142 assay (≥1% IC) or Dako 28-8 assay (PD-L1 combined positive score (CPS) ≥1, ≥3, ≥5, ≥10 and ≥20) (Supplementary Fig. 4). The highest overall percentage agreement of 80.6% was observed between SP142 ≥1% IC and 28-8 CPS ≥ 5 (Fig. 4a). pCR and RCB 0 or I rates were increased in patients with PD-L1+ tumors as measured by both SP142 (≥1% IC) and 28-8 CPS (≥1); the benefit was greater with increasing CPS cutoffs (Fig. 4c and Supplementary Figs. 5 and 6).

The prevalence of biomarker-positive populations at baseline stratified by percentage of stromal tumor-infiltrating lymphocytes (sTILs) at various cutoffs, compared with those by PD-L1 SP142 at 1% IC, is shown in Supplementary Fig. 7. Median and mean sTIL levels were 1% and 14.2% (s.d., 24.16), respectively, and the prevalence of sTIL positivity was balanced across the treatment groups. Defining sTIL-positive patients as those with detectable sTILs (>1%), the overall percentage agreement between sTIL detection and various PD-L1 by immune cell or CPS cutoffs ranged between 67.0% and 72.4% (Fig. 4b). pCR and RCB 0 or I rates with nivolumab versus placebo increased in patients with higher sTIL levels (Figs. 2d,f and 4d). When both sTIL and PD-L1 assays were used, the highest pCR rates were observed in patients in whom both sTIL and PD-L1 expression were considered positive, but notably, there was also nivolumab benefit seen for patients with discordance between PD-L1 <1% IC and sTIL+ (Supplementary Fig. 8).

pCR and RCB 0 or I rates were higher in patients whose tumors had lower ER (<50%) and/or progesterone receptor expression (<10%) than in patients whose tumors had higher ER or progesterone receptor expression (Supplementary Figs. 9 and 10). No association between nivolumab benefit and the Ki67 index was observed (Supplementary Fig. 11).

In a multivariable analysis of pCR by biomarker subgroups, including prognostic clinicopathological features and key biomarkers, sTIL percentage (>1% or ≥5%) and PD-L1 (defined as IC ≥1% or CPS ≥3) were independently associated with nivolumab efficacy (Supplementary Figs. 12a,b and 13a,b).

### Safety

The safety analysis is based on the safety population (*N* = 517; 262 patients in the nivolumab arm and 255 patients in the placebo arm). In the neoadjuvant treatment phase, a similar proportion of patients in the nivolumab versus placebo arms experienced adverse events (AEs) (98.5% versus 98.4%) and treatment-related AEs of any grade (95.0% versus 91.8%). The most frequently reported treatment-related AEs were alopecia (48.9% versus 48.2%), nausea (45.0% versus 36.9%), anemia (36.3% versus 29.4%) and fatigue (31.7% versus 25.5%) in the nivolumab versus placebo arms, respectively. Grade 3 or 4 AEs were reported in 42.0% versus 38.4% of patients in the nivolumab versus placebo arm, respectively. Grade 3 or 4 treatment-related AEs were reported in 35.1% versus 32.5% of patients in the nivolumab versus placebo arms, respectively (Table 2). Serious AEs (22.9% versus 12.9%) and treatment-related serious AEs (14.5% versus 8.2%), as well as AEs leading to discontinuation (11.5% versus 2.7%) and treatment-related AEs leading to discontinuation (10.7% versus 2.7%), were reported more frequently with nivolumab than with placebo.

**Table 2:** Neoadjuvant safety summary (safety population)^a^

AE	Nivolumab plus neoadjuvant CT (*n* = 262)		Placebo plus neoadjuvant CT (*n* = 255)	
	Any grade *n* (%)	Grade 3 or 4 *n* (%)	Any grade *n* (%)	Grade 3 or 4 *n* (%)
AE	258 (98.5)	110 (42.0)	251 (98.4)	98 (38.4)
TRAE	249 (95.0)	92 (35.1)	234 (91.8)	83 (32.5)
SAE	60 (22.9)	43 (16.4)	33 (12.9)	28 (11.0)
TRSAE	38 (14.5)	34 (13.0)	21 (8.2)	20 (7.8)
AE leading to discontinuation	30 (11.5)	18 (6.9)	7 (2.7)	6 (2.4)
TRAE leading to discontinuation	28 (10.7)	17 (6.5)	7 (2.7)	6 (2.4)
TRAE^b^				
Alopecia^c^	128 (48.9)	3 (1.1)	123 (48.2)	5 (2.0)
Nausea	118 (45.0)	0	94 (36.9)	2 (0.8)
Anemia	95 (36.3)	15 (5.7)	75 (29.4)	6 (2.4)
Fatigue	83 (31.7)	5 (1.9)	65 (25.5)	2 (0.8)
Diarrhea	57 (21.8)	4 (1.5)	58 (22.7)	1 (0.4)
Peripheral neuropathy	52 (19.8)	3 (1.1)	37 (14.5)	1 (0.4)
Increased ALT	44 (16.8)	6 (2.3)	32 (12.5)	8 (3.1)
Increased AST	45 (17.2)	6 (2.3)	28 (11.0)	2 (0.8)
Neutropenia	44 (16.8)	16 (6.1)	42 (16.5)	25 (9.8)
Vomiting	40 (15.3)	1 (0.4)	25 (9.8)	2 (0.8)
Endocrine IMAEs^d,e^				
Hypothyroidism/thyroiditis	39 (14.9)	0	3 (1.2)	0
Adrenal insufficiency	15 (5.7)	4 (1.5)	1 (0.4)	0
Hyperthyroidism	15 (5.7)	0	1 (0.4)	0
Hypophysitis/hypopituitarism	5 (1.9)	1 (0.4)	0	0
Diabetes mellitus	1 (0.4)	0	0	0
Nonendocrine IMAEs^d,e^ where immunomodulation was initiated				
Rash	15 (5.7)	4 (1.5)	12 (4.7)	1 (0.4)
Hepatitis	13 (5.0)	8 (3.1)	3 (1.2)	2 (0.8)
Pneumonitis	8 (3.1)	4 (1.5)	2 (0.8)	0
Hypersensitivity	10 (3.8)	0	3 (1.2)	0
Nephritis/renal dysfunction	2 (0.8)	1 (0.4)	0	0
Diarrhea/colitis	1 (0.4)	0	1 (0.4)	1 (0.4)

^a^Database lock: 20 March 2024.

^b^Events reported between the first dose and 30 days after the last dose of neoadjuvant therapy for patients who did not go on to adjuvant therapy or before adjuvant therapy for patients who started adjuvant therapy. The events shown are the 10 most frequent in the nivolumab arm.

^c^Alopecia is likely to have been under-reported. Common Toxicity Criteria for Adverse Events grading for alopecia consists of only grade 1 or 2; the grade 3 or 4 alopecia reported in this study was reported incorrectly.

^d^Events reported between the first dose and 100 days after the last dose of neoadjuvant therapy for patients who did not go on to adjuvant therapy or before adjuvant therapy for patients who started adjuvant therapy.

^e^Immune-mediated adverse events (IMAEs) are specific events, regardless of causality, that were considered as potentially immune-mediated by the investigator with no clear alternate etiology, occurred within 100 days of the final dose, and were treated with immune-modulating medication (except for endocrine IMAEs, which do not require immune-modulating medication use).

ALT, alanine transaminase; AST, aspartate transaminase; SAE, serious adverse event; TRAE, treatment-related adverse event; TRSAE, treatment-related serious adverse event.

In the neoadjuvant treatment phase, there were three (1.1%) grade 5 treatment-unrelated events in the nivolumab arm (one due to COVID-19; two due to pulmonary embolism, of which one occurred within a week postrecovery from COVID-19) and none in the placebo arm. In addition, two further deaths in the nivolumab arm were deemed related to study drug toxicity, although not reported as grade 5 (because of the extended time interval between AE onset and death): pneumonitis (61 days after final dose of neoadjuvant treatment) and hepatitis (51 days after final dose of neoadjuvant treatment); no deaths due to study drug toxicity were reported with placebo.

AEs during the neoadjuvant treatment phase that required immune-modulating medication occurred in 135 (51.5%) and 87 (34.1%) patients in the nivolumab and placebo arms, respectively. AEs of special interest occurred in three (1.1%) patients in the nivolumab arm and no patients in the placebo arm; these events were grade 3 or 4 Guillain–Barré syndrome (*n* = 1, 0.4%), grade 3 or 4 myocarditis (*n* = 1, 0.4%) and grade ≤2 autoimmune neuropathy (*n* = 1, 0.4%).

The mean cumulative dose and relative dose intensity of each CT drug were similar in both treatment arms.

## Discussion

In the CheckMate 7FL study, we investigated whether the addition of nivolumab to anthracycline and taxane neoadjuvant CT could significantly increase pCR rates in newly diagnosed early-stage, high-risk, high-grade ER+/HER2− BC. The study met its primary endpoint, with a significantly higher rate of pCR in the nivolumab arm versus placebo. RCB 0 or I rates were also improved in the nivolumab versus placebo arm. These findings were predominantly driven by the PD-L1+ subpopulation, in which an absolute difference of more than 20% was seen with the addition of nivolumab to the neoadjuvant CT. This observation differed from that in early-stage TNBC, where the effect was independent of PD-L1 expression^18^. Although the reasons for this are unclear, TNBC is known to be more molecularly heterogeneous than ER+ BC, potentially resulting in a single core biopsy unlikely to encapsulate heterogenous PD-L1 expression^19,20^. The median follow-up remains too short in this analysis to make any conclusions about EFS, although notably there were no early non-BC-related deaths. However, achievement of a pCR and/or a RCB of 0 or I is associated with improved long-term outcomes in ER+/HER2− BC^21,22^. Translation of improvements in pCR rates into EFS improvements varies across different clinical trials. Very few early trials were adequately powered to assess both endpoints; however, overall, almost all combination chemotherapies that improved pCR rates (by incorporating a taxane-based, carboplatin-based, HER2-targeted therapy or pembrolizumab) also improved EFS in BC studies^23,24^. It is also becoming increasingly clear that different neoadjuvant regimens result in different distributions of RCB, and therapies that shift the entire spectrum of RCB to smaller values may have a greater impact on EFS than therapies that improve pCR rates by moving minimal residual cancers to the pCR category^25^.

Our results are consistent with those of the KEYNOTE-756 study^26,27^, which investigated pembrolizumab in the same patient setting. In KEYNOTE-756, improved pCR rates were also seen in the setting of increasing PD-L1 expression but only at the higher levels using the 22C3 pharmDx CPS (CPS ≥ 10) assay. Results from CheckMate 7FL consolidate the benefit of adding an immune checkpoint inhibitor to neoadjuvant CT in this BC subtype and context, and longer follow-up will indicate whether these pCRs translate into greater EFS benefit for all or just for patients with PD-L1+ tumors. Notably, whereas previous studies have shown that addition of programmed death 1 inhibition in TNBC led to small increases in pCR rates, including in patients with low PD-L1 expression, significant EFS benefit was observed^28,29^. Overall, these results represent a new milestone in the neoadjuvant treatment of ER+/HER2− BC, because there have been intensive but thus far unsuccessful efforts to improve pCR rates in this patient population.

One important strength of CheckMate 7FL is that three immune assays were evaluated in a phase III trial population. Increases in pCR rates with the addition of nivolumab were also observed for sTIL increases from as little >1%, which was the median sTIL level in this patient population. Moderate concordance between the SP142, 28-8 pharmDx CPS and sTIL assays was observed. Interestingly, although pCR rates were highest with the addition of nivolumab when the assays agreed on sTIL and PD-L1 positivity, patients with discordant assay results still derived benefit. These data have important implications for patients with BC, and suggest that the use of multiple assays may be best to identify all patients who could benefit from neoadjuvant immunotherapy in this subtype, although sTIL may be the most pragmatic and globally accessible biomarker because it can be evaluated on standard hematoxylin and eosin-stained slides^30,31^. Analysis of other exploratory biomarkers for patient stratification is ongoing.

Analyses of pCR rates by ER and progesterone receptor levels confirm that patients with ER and/or progesterone receptor levels <10% have greater benefit with the addition of nivolumab than patients with ER and/or progesterone receptor levels ≥10%. Notably, we observed this effect also in the setting of ER ≤50%. Although this remains to be further validated, it suggests that patients with lower ER and progesterone receptor levels may be treated similarly to patients with early TNBC. Previous research has shown that tumors with lower ER levels harbor more sTILs and CD8^+^ T cells, with higher PD-L1 expression, and are more similar to TNBC with regard to immune-related signatures^32^.

Safety was consistent with the known safety profiles, with no change in the feasibility of surgery following the addition of nivolumab to CT. However, it is important to note that two treatment-related deaths were observed in the nivolumab arm.

The key strengths of this study are: its inclusion of a high-risk population, the majority of whom were node-positive and grade 3; evaluation of response in a PD-L1+ population as a key secondary endpoint; and comprehensive biomarker data, including evaluation of response in a sTIL-high population, presented in the early BC setting. Limitations include the major protocol amendment that significantly reduced the sample size and/or number of events and follow-up time resulting in EFS being designated as an exploratory endpoint.

In conclusion, adding nivolumab to neoadjuvant anthracycline and taxane-based CT in high-risk, early-stage ER+/HER2− BC significantly increased the pCR rate. These findings reshape our understanding of this disease in the context of T cell immunosurveillance and immunotherapy response in luminal disease. Patients with higher levels of sTIL or PD-L1 expression experienced higher pCR rates, potentially setting a new standard for future neoadjuvant treatment studies in this subset. Biomarker analyses aim to uncover the biological drivers behind the robust immune responses to the addition of immunotherapy to CT observed in ER+ BC, which could help further refine and personalize immunotherapeutic approaches for this disease.

## Methods

### Patients

Eligible patients had newly diagnosed ER+/HER2− BC, with a confirmed primary tumor and node categories of tumors sized 2–5 cm and cN1–cN2 or cT3–cT4 and cN0–cN2; grade 3 disease or grade 2 disease with ER expression of 1 to ≤10%; adequate organ function; tissue available for biomarker assessment; and Eastern Cooperative Oncology Group performance status 0–1. Patients were eligible irrespective of PD-L1 status. Multifocal tumors (two or more foci of cancer in the same breast quadrant) were permitted if the largest lesion was at least 2 cm and designated as the target lesion. Patients with mixed ductal and lobular carcinoma were eligible. Patients were excluded if they had multicentric BC, a history of ipsilateral invasive BC, evidence of metastatic disease, had received any previous treatment for the currently diagnosed BC or had received immunotherapy previously.

### Trial design and treatments

CheckMate 7FL was a prospective, randomized, multicenter, double-blind, placebo-controlled phase 3 trial (ClinicalTrials.gov identifier: NCT04109066), originally with co-primary endpoints of pCR and EFS, which were centrally assessed. Following the approval of adjuvant abemaciclib for high-risk primary ER+/HER2, the primary endpoint was amended to pCR alone, making enrollment and assessment of EFS challenging to complete. The combination of abemaciclib with nivolumab was expected to result in a high rate of withdrawals because of safety concerns around combining a CDK4/6 inhibitor with an anti-programmed death 1 agent^33,34^. In the neoadjuvant phase, patients were randomized 1:1 to receive either nivolumab 360 mg or placebo every 3 weeks with weekly paclitaxel for 12 weeks. This was followed by nivolumab (either 360 mg every 3 weeks or 240 mg every 2 weeks) in combination with anthracycline and cyclophosphamide, or placebo in combination with anthracycline and cyclophosphamide; the anthracycline and cyclophosphamide dosing frequency was determined by the investigator. All patients who remained operative candidates underwent surgery of the breast and axilla (per local standards) within 4 weeks of completing the neoadjuvant treatment phase. Per the protocol amendment, the study was unblinded in the adjuvant phase, and patients received nivolumab 480 mg with investigator’s choice of ET (tamoxifen, letrozole, anastrozole or exemestane, with or without ovarian function suppression) for up to seven cycles.

Randomization was stratified per interactive response technology by the proportion of PD-L1-expressing immune cells (percentage of immune cells by VENTANA PD-L1 SP142 immunohistochemistry, cutoff at 1%), tumor grade (2 or 3), pathologically confirmed axillary nodal status (positive on pathological review or negative on radiographic and/or pathologic review) and anthracycline dosing frequency (every 3 weeks or every 2 weeks). Before the study was initiated, each participant received log-in information and directions on accessing the interactive response technology. Each participant was assigned a unique number after signing the informed consent form. Participant numbers were used on all participants’ study information. Participant numbers were not reassigned. An interactive response technology was used to manage participant randomization. The investigator or designee registered the participant for enrollment by following the enrollment procedures established by the sponsor.

### Endpoints

The primary endpoint was pCR (ypT0/is, ypN0) in the mITT population. Initially, EFS was a co-primary endpoint; however, following the decision to discontinue enrollment in the study in April 2022 because of the rapidly changing treatment landscape, the primary endpoint of the trial was updated to focus solely on pCR, and EFS was changed to an exploratory endpoint because the total number of enrolled patients and events was too low and updated follow-up time was too short to provide sufficient power for comparison. Consequently, follow-up was reduced to 1 year post-surgery for all patients, and the adjuvant phase became open label.

Another key change to the study after discontinuation of enrollment in April 2022 was the evaluation of pCR in the PD-L1+ population by VENTANA SP142 % IC as a secondary endpoint. Other secondary endpoint included RCB 0 or I rates in the mITT and PD-L1+ populations. Pathological response was assessed, and RCB score calculated by local pathologists. The RCB score combined tumor size, tumor cellularity and nodal involvement into a single continuous score that was grouped into four classes, namely, RCB score of 0 (that is, pCR), and I, II and III, which corresponded to increasingly larger residual cancer and worse recurrence-free survival^17^. Safety and tolerability were assessed during the neoadjuvant and adjuvant phases in all patients who received at least one dose of study drug. Prespecified exploratory endpoints included association of efficacy outcomes with biomarker status based on PD-L1 CPS, sTILs, levels of estrogen/progesterone receptors and Ki67 index.

### Study assessments

pCR was assessed post-neoadjuvant (yp) treatment and was defined as no invasive residual disease in breast and lymph nodes (ypT0/is, ypN0) by a local pathologist. AEs were monitored throughout the trial and for 30 days after the discontinuation of study treatment (90 days for serious AEs) and graded according to Common Toxicity Criteria for Adverse Events v.5.0 of the National Cancer Institute. Safety was assessed at 30 days and 100 days after the final dose, and long-term follow-up was up to 12 months after surgery. Biomarker analyses included centrally reviewed PD-L1 expression and percentage of sTILs. PD-L1 was evaluated by qualitative immunohistochemistry on immune cells with the VENTANA SP142 assay (Roche Diagnostics) and PD-L1 CPS with the 28-8 pharmDx assay (Agilent). The percentage of sTILs was quantified on a hematoxylin and eosin-stained slide according to established guidelines^30^. In this study, an sTIL of 1% was the lowest possible value and indicated a lack of detectable sTILs. The SP142 % IC assay and the 28-8 pharmDx CPS were used to evaluate the variation between assays, as well as to determine PD-L1 expression in tumor-infiltrating ICs versus both immune and tumor cells. ER and Ki67 expression were centrally evaluated using Agilent MIB-Dako pharmDx immunohistochemistry. Progesterone receptor immunohistochemistry levels were evaluated from local pathology testing. Other recorded patient and disease characteristics included tumor grade, axillary nodal status, disease stage, Ki67 index, menopausal status and age.

### Statistical analyses

Based on the normal approximation to the binomial, a sample size of 521 patients in the intent-to-treat population would yield approximately 87% power (two-sided alpha of 0.05) to detect a difference of 10% in pCR rates between treatment arms, assuming a 12% pCR rate in the control arm. Because of the sponsor’s decision to close all sites in Russia after the Ukraine–Russia geopolitical conflict began, 11 patients were excluded owing to insufficient follow-up for pCR assessment, with a small impact on the study power (86%). This resulted in a mITT population size of 510 patients. Strata-adjusted difference in pCR rate between the two arms was analyzed with the stratified Cochran–Mantel–Haenszel method of weighting with a two-sided alpha level of 0.05. Strata-adjusted OR was assessed with the Mantel–Haenszel method. The cutoffs used for sTIL and PD-L1 expression by SP142 or 28-8 pharmDx CPS were predefined for this study.

CI values for pCR and RCB 0 or I rates were evaluated using the Clopper–Pearson method. The unweighted differences in pCR and RCB 0 or I rates between treatment arms in different patient subgroups were calculated along with the corresponding 95% two-sided CIs using the Newcombe method without continuity correction. Exploratory multivariable analyses were conducted using logistic regression to evaluate the association of biomarkers and other baseline characteristics with pCR. Biomarkers included in the multivariable analyses were calculated as either categorical (PD-L1 expression ≥1% by SP142 or 28-8 pharmDx CPS ≥3 with sTIL cutoffs of >1 or ≥5%) or continuous variables. Other baseline characteristics in the multivariable analyses were stage III disease, negative nodal status, ER expression ≥10%, postmenopausal status and age ≥65 years. The CIs for the secondary and exploratory analyses were for descriptive purposes and, therefore, no adjustments were made for multiplicity.

The sex of patients enrolled in the trial was self-reported and data for gender were not collected. No analyses by sex or disaggregated data are presented because doing so would result in the presentation of potentially identifying information.

### Trial oversight

This trial was developed and overseen by an academic steering committee and employees of the sponsor (Bristol Myers Squibb). An external, independent data monitoring committee provided oversight of safety and efficacy considerations during the study. The trial protocol and amendments were approved by the appropriate ethics body at each participating site. All patients provided written informed consent. All authors confirm that the trial was conducted with respect to the standards of Good Clinical Practice. All authors had access to the data and participated in the writing and reviewing of this manuscript. The first draft of the manuscript was written by the first author with editorial assistance provided by a medical writer employed by the sponsor. All authors reviewed and participated in drafting the manuscript and all authors approved the submitted draft and can vouch for the accuracy and completeness of the data.

### Reporting summary

Further information on research design is available in the Nature Portfolio Reporting Summary linked to this article.

## Online content

Any methods, additional references, Nature Portfolio reporting summaries, source data, extended data, supplementary information, acknowledgements, peer review information; details of author contributions and competing interests; and statements of data and code availability are available at 10.1038/s41591-024-03414-8.

## Supplementary information

Supplementary Information Supplementary Table 1 and Figs. 1–13.

Reporting Summary

## Data Availability

Bristol Myers Squibb will honor legitimate requests for clinical trial data from qualified researchers with a clearly defined scientific objective. Bristol Myers Squibb will consider data sharing requests for Phase II–IV interventional clinical trials that completed on or after 1 January 2008. In addition, primary results from these trials must have been published in peer-reviewed journals and the medicines or indications approved in the US, EU and other designated markets. Sharing is also subject to protection of patient privacy and respect for the patient’s informed consent. Data considered for sharing may include nonidentifiable patient-level and study-level clinical trial data, full clinical study reports and protocols.
