# Supplementary material for: Neoadjuvant nivolumab and chemotherapy in early estrogen receptor-positive breast cancer: a randomized phase 3 trial
Source: Nat Med. 2025 Jan 21;31(2):433–41. doi: 10.1038/s41591-024-03414-8 (PMC11835735; doi:10.1038/s41591-024-03414-8)
Supplement: Supplementary file 1 — Supplementary Table 1 and Figs. 1–13. [file 41591_2024_3414_MOESM1_ESM.pdf]

nature medicine

Supplementary information https://doi.org/10.1038/s41591-024-03414-8

Neoadjuvant nivolumab and chemotherapy in early estrogen receptor-positive breast cancer: a randomized phase 3 trial

In the format provided by the authors and unedited

Supplementary appendix

Table of Contents

Table S1. Surgery summary in all randomized patients. 2

Figure S1. Subgroup analyses of residual cancer burden 0/I rates. 3

Figure S2. RCB class distribution in the (A) modified intent-to-treat and (B) PD-L1 ≥1% (SP142) populations. 4

Figure S3. Event-free survival. 6

Figure S4. Prevalence of baseline PD-L1 expression by SP142 IC% and 28-8 CPS at various cutoffs. 7

Figure S5. pCR rate by PD-L1 status as determined by the SP142 (IC%) and 28-8 CPS (cutoffs 1–20) assays. 8

Figure S6. RCB 0/I rate by PD-L1 status as determined by the SP142 (IC%) and 28-8 CPS (cutoffs 1–20) assays. 10

Figure S7. Prevalence of baseline PD-L1 expression by SP142 IC% and sTIL at various cutoffs. 12

Figure S8. Interaction between PD-L1 and sTIL levels on pathological complete response rates as determined by the SP142 (% IC) and 28-8 CPS assays and percentage of sTIL at sTIL cutoffs of 1% (A) and 5% (B). 13

Figure S9. pCR and RCB 0/I rates by tumor ER expression. 15

Figure S10. pCR and RCB 0/I rates by tumor PR expression. 16

Figure S11. pCR and RCB 0/I rates by Ki67 index. 17

Figure S12. Multivariable analysis of pCR by biomarkers: (A) PD-L1 on ICs with 5% sTIL cutoff, (B) PD-L1 CPS with 5% sTIL cutoff, (C) PD-L1 on ICs with 1% sTIL cutoff, (D) PD-L1 CPS with 1% sTIL cutoff, and other baseline disease characteristics. 18

Figure S13. Multivariable analysis of pCR by biomarker: (A) PD-L1 on IC and (B) PD-L1 CPS, and other baseline disease characteristics. 20

Table S1. Surgery summary in all randomized patients.[a]

n, (%)	Nivolumab plus neoadjuvant chemotherapy n = 263	Placebo plus neoadjuvant chemotherapy n = 258
**Patients who underwent surgery**	233 (89)	236 (91)
Breast-conserving surgery	99 (38)	101 (39)
Mastectomy	134 (51)	135 (52)
**Patients who underwent surgery but with delay**	67 (25)	45 (17)
AE[b]	24 (9)	6 (2)
Logistical issues	29 (11)	28 (11)
Patient decision	4 (2)	1 (<1)
Other[c]	10 (4)	10 (4)
**Patients who did not undergo surgery[d]**	30 (11)	22 (9)

[a]AE denotes adverse event and COVID-19.
[b]Included immune-mediated AEs, infection, cytopenia, and other.
[c]Included insurance/administrative and COVID-19 pandemic-related issues.
[d]The most common reasons for not undergoing surgery were withdrawal of consent and enrollment at Russian sites that had closed prior to surgery. Other reasons included treatment discontinuation due to study drug toxicity/AE/investigator/patient decision, death, not meeting study criteria after randomization, disease progression, emergency unblinding, and moving out of country.

Figure S1. Forest plot of proportion of patients with RCB 0/I rates in the nivolumab plus neoadjuvant chemotherapy and placebo plus neoadjuvant chemotherapy arms by subgroup analyses.

	n	RCB 0/I Rate, % (95% CI): Nivolumab Plus Neoadjuvant Chemotherapy	RCB 0/I Rate, % (95% CI): Placebo Plus Neoadjuvant Chemotherapy	Unweighted RCB 0/I Rate Difference % (95% CI)
**Overall**	510	30.7 (25.2–36.8)	21.3 (16.5–26.9)	9.4 (1.8–16.9)
**PD-L1 on ICs by SP142 (IRT)**				
< 1%	338	18.3 (12.8–25.0)	18.9 (13.3–25.7)	−0.6 (−8.9–7.7)
≥ 1%	172	54.5 (43.6–65.2)	26.2 (17.2–36.9)	28.4 (13.7–41.3)
**Axillary nodal status (IRT)**				
Positive	406	30.2 (24.0–37.0)	18.4 (13.3–24.5)	11.8 (3.5–20.0)
Negative	104	32.7 (20.3–47.1)	32.7 (20.3–47.1)	0.0 (−17.6–17.6)
**Baseline anatomical stage defined by TNM classification**				
Stage I	1	NA	0.0 (0.0–97.5)	
Stage II	273	29.6 (22.1–38.1)	23.9 (17.1–31.9)	5.7 (−4.8–16.1)
Stage III	223	32.2 (23.9–41.4)	18.1 (11.3–26.8)	14.1 (2.6–24.9)
Stage IV[a]	2	NA	0.0 (0.0–84.2)	
Not assigned	9	25.0 (0.6–80.6)	20.0 (0.5–71.6)	
Not reported	2	NA	50.0 (1.3–98.7)	
**Age**				
< 50	241	28.7 (20.9–37.6)	20.2 (13.4–28.5)	8.5 (−2.4–19.1)
≥ 50	269	32.6 (24.8–41.2)	22.4 (15.6–30.4)	10.2 (−0.5–20.6)
**Ethnic background**				
White	384	31.6 (25.2–38.6)	21.3 (15.7–27.8)	10.4 (1.5–19.0)
Black or African American	14	33.3 (4.3–77.7)	25.0 (3.2–65.1)	
Asian	42	16.7 (4.7–37.4)	11.1 (1.4–34.7)	5.6 (−18.3–26.3)
American Indian or Alaska Native	35	38.9 (17.3–64.3)	29.4 (10.3–56.0)	9.5 (−20.7–37.2)
Native Hawaiian or Other Pacific Islander	5	0.0 (0.0–84.2)	33.3 (0.8–90.6)	
Other	29	36.4 (10.9–69.2)	22.2 (6.4–47.6)	14.1 (−17.1–45.3)
Not reported	1	NA	0.0 (0.0–97.5)	
**AC dose-frequency chemotherapy regimen (IRT)**				
Q2W	266	32.6 (24.7–41.3)	24.6 (17.6–32.8)	7.9 (−2.9–18.6)
Q3W	244	28.8 (21.1–37.6)	17.6 (11.3–25.7)	11.2 (0.5–21.4)

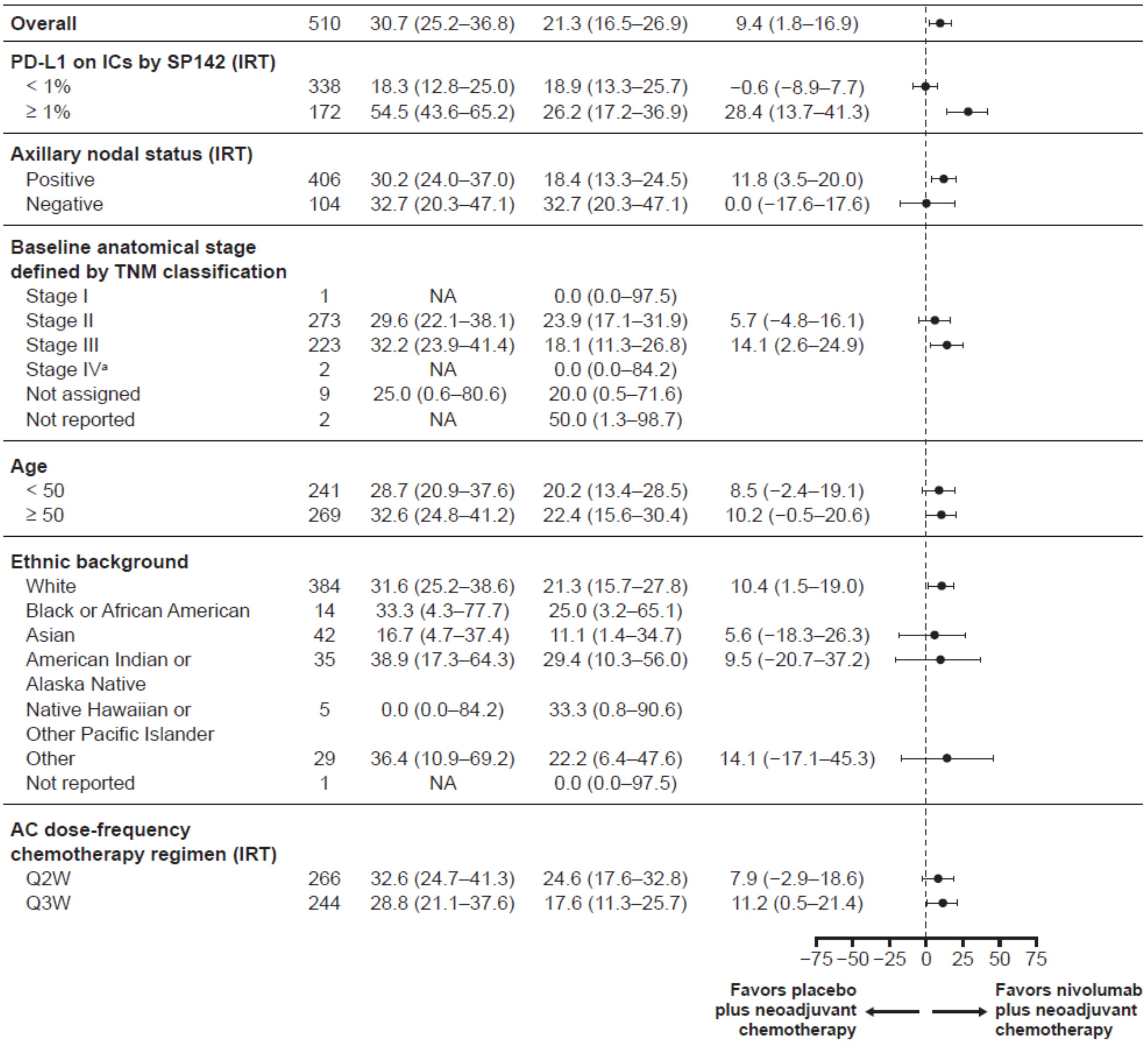

Data are presented as percentages with error bars showing the 95% CI around the observed proportion of patients in the treatment arm. The CIs for each treatment arm were calculated using the Clopper–Pearson method and CIs for differences between treatment arms were calculated using the Newcombe method without continuity correction. RCB 0/I rate rate difference was not computed for subsets with fewer than 10 patients per treatment arms. [a]The two patients who were initially categorized as having stage IV disease were deemed eligible and later re-categorized as having stage II disease. AC, anthracycline; CI, confidence interval; IC, immune cell; IRT, interactive response technology; n, total number of patients in subgroup; NA, not available; PD-L1, programmed death ligand 1; Q2W, every two weeks; Q3W, every 3 weeks; TMN, staging system (T, size and extent of primary tumor; N, extent of spread to the lymph nodes; M, presence of metastasis); RCB, residual cancer burden. Database lock: April 14, 2023.

Figure S2. RCB class distribution (0, I, II, and III) in the nivolumab plus neoadjuvant chemotherapy and placebo plus neoadjuvant chemotherapy arms in the modified intent-to-treat (A) and PD-L1 ≥1% (SP142) populations (B).

A

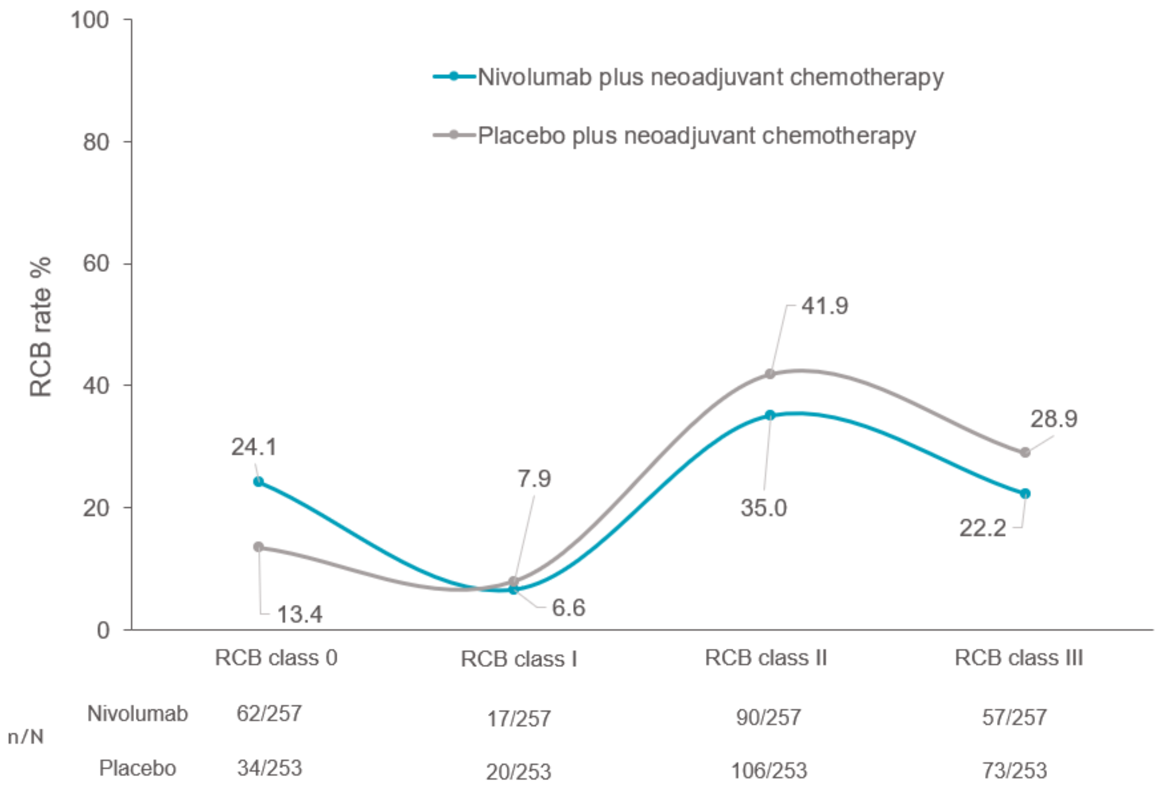

B

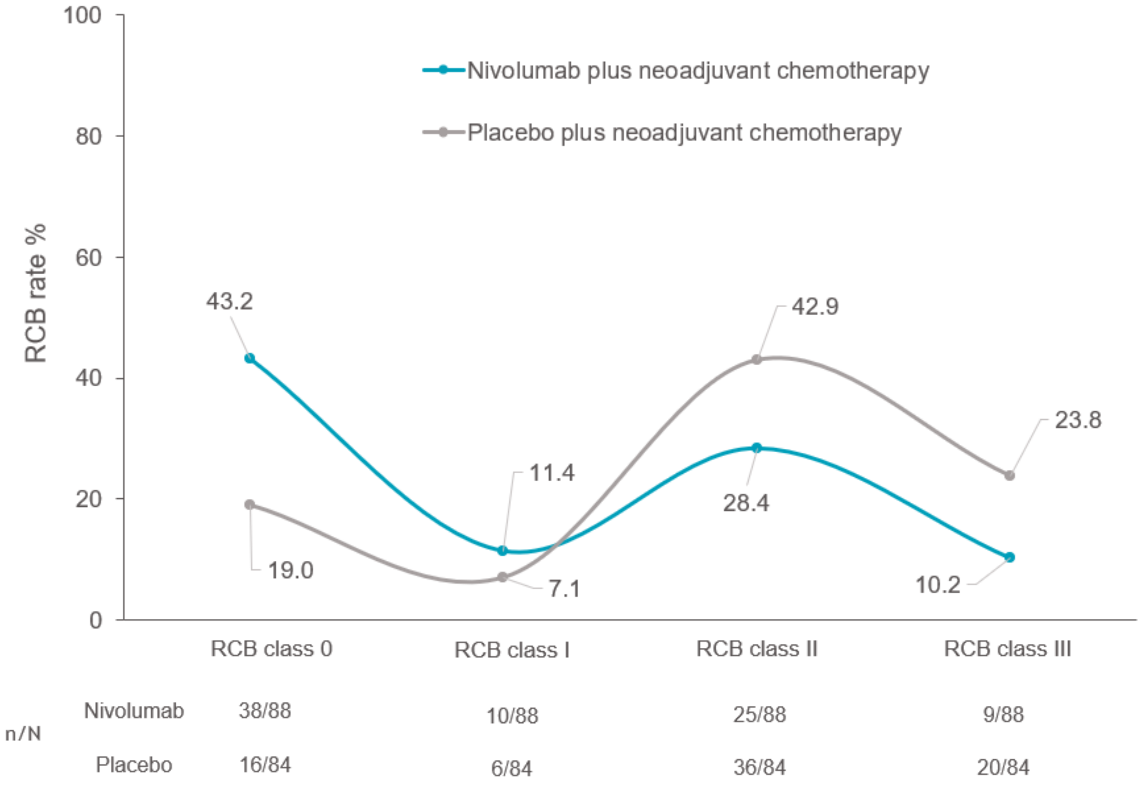

The number of patients with qualifying data and the total number of patients in each subgroup is shown below each chart (n/N). Does not include unreported data for 31 and 20 patients in the nivolumab and placebo arms, respectively for the mITT population, and 6 and 6 patients in the nivolumab and placebo arms, respectively for the PD-L1+ population. n, number of patients with RCB; N, total number of patients in subgroup; PD-L1, programmed death ligand 1; RCB, residual cancer burden; SP142 VENTANA®, PD-L1 SP142 assay.

Figure S3. Event-free survival in the nivolumab plus neoadjuvant chemotherapy and placebo plus neoadjuvant chemotherapy arms.

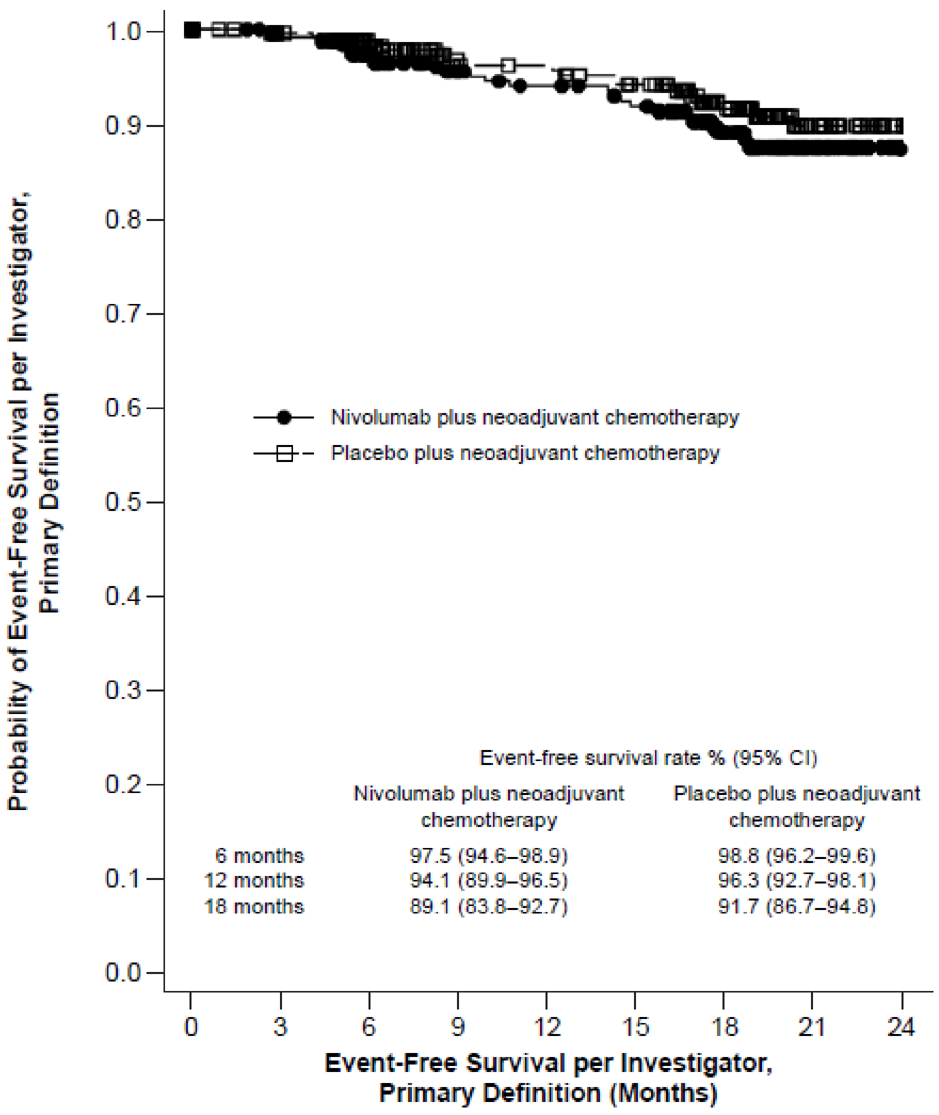

Number of subjects at risk

Nivolumab plus neoadjuvant chemotherapy	257	244	213	188	181	174	135	73	45
Placebo plus neoadjuvant chemotherapy	253	242	218	184	181	171	134	81	59

Nivolumab plus neoadjuvant chemotherapy, number of events: 24/257; median and 95% CI, NA
Placebo plus neoadjuvant chemotherapy, number of events: 23/253; median and 95% CI, NA (35.35, NA)

The CIs for event-free survival (exploratory analyses) were for descriptive purposes and, therefore, no adjustments were made for multiplicity. CI, confidence interval; NA, not available (not reached). Database lock: March 20, 2024.

Figure S4. Prevalence of baseline PD-L1 expression in the nivolumab plus neoadjuvant chemotherapy and placebo plus neoadjuvant chemotherapy arms by SP142 IC% and 28-8 CPS at various cutoffs.

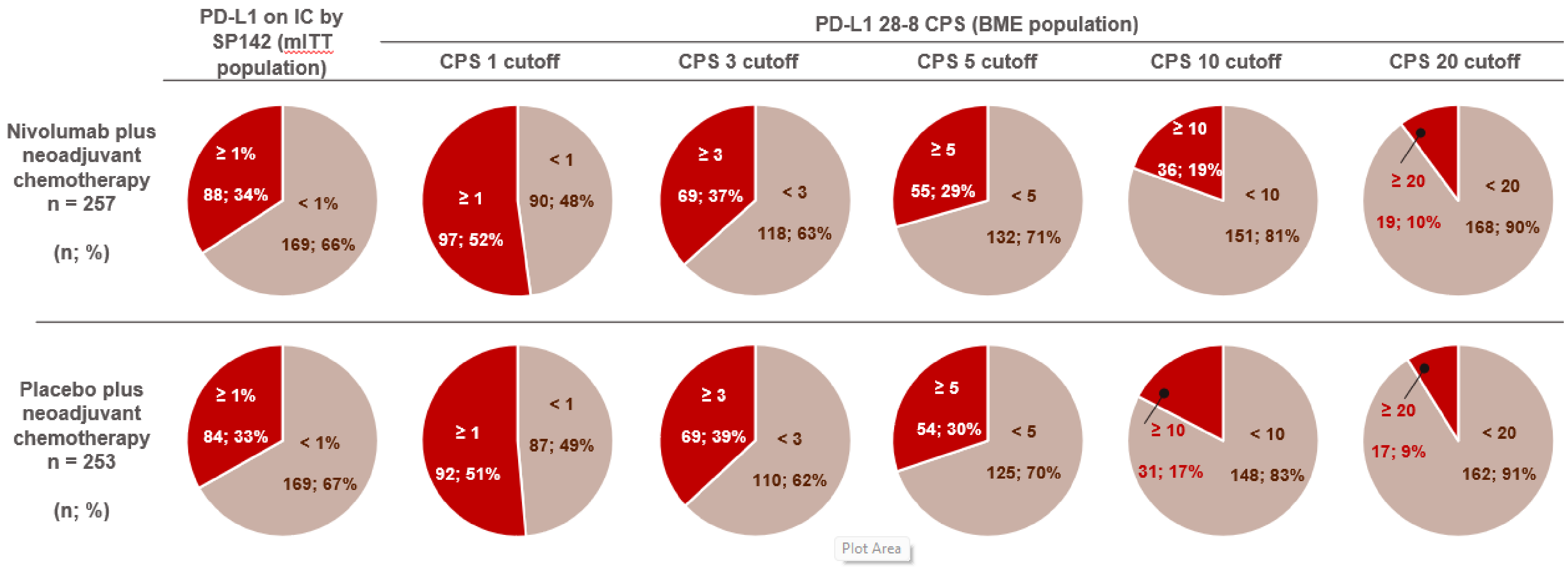

28-8 CPS, Dako 28-8 assay using the CPS algorithm; BME, biomarker evaluable; CPS, combined positive score; IC, immune cell; mITT, modified intent-to-treat; n, number of patients with PD-L1 expression; PD-L1, programmed death ligand 1; SP142 VENTANA® PD-L1 SP142 assay. Database lock: March 20, 2024.

Figure S5. Proportion of patients with pCR in the nivolumab plus neoadjuvant chemotherapy and placebo plus neoadjuvant chemotherapy groups by PD-L1 status as determined by the SP142 (IC% < 1% and ≥ 1%) and 28-8 CPS (cutoffs 1–20) assays. Data are presented as percentages with error bars showing the 95% CI around the observed proportion of patients in the treatment arm. The CIs for each treatment arm were calculated using the Clopper–Pearson method and CIs for differences between treatment arms were calculated using the Newcombe method without continuity correction. The number of patients with pCR and the total number of patients in each subgroup is shown under each bar as n/N.

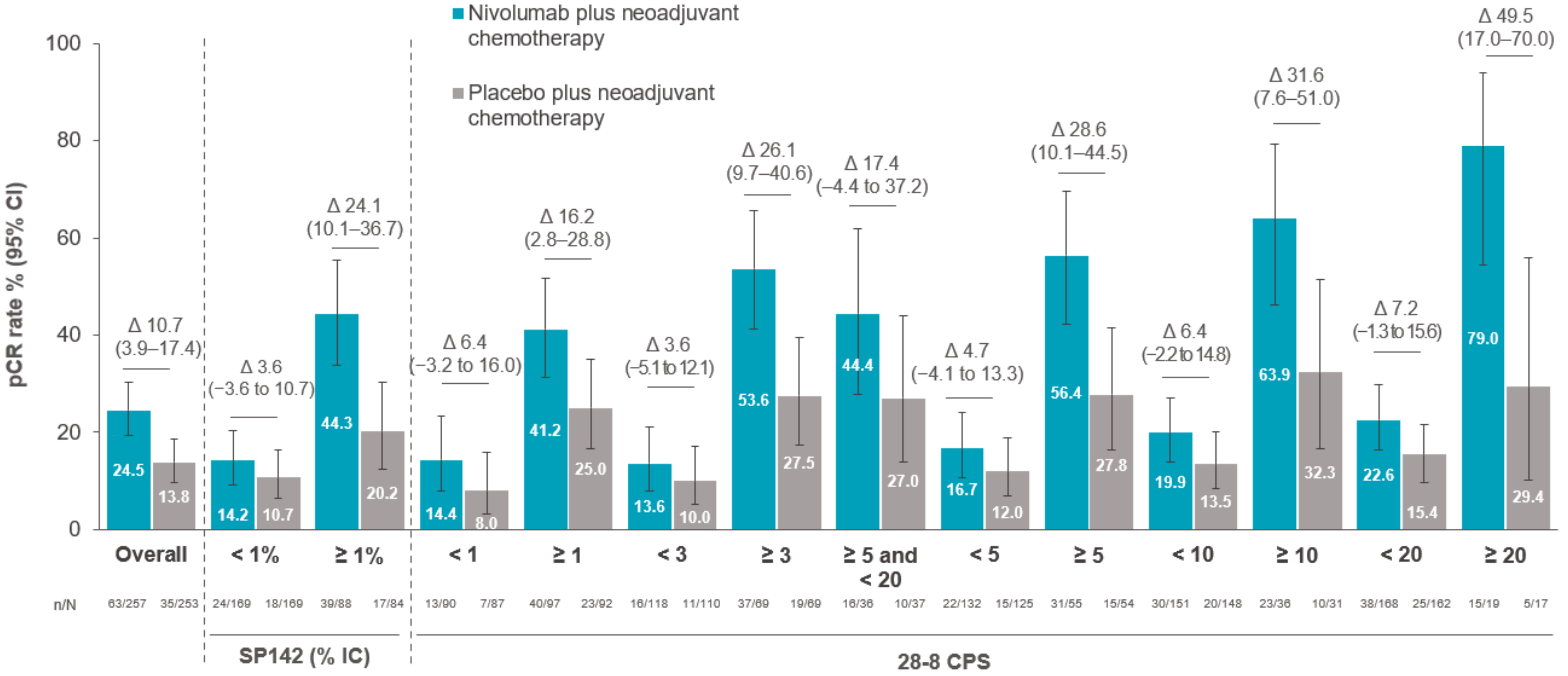

28-8 CPS, Dako 28-8 assay using the CPS algorithm; CI, confidence interval; CPS, combined positive score; IC, immune cell; n, number of patients with pCR; N, number of patients in the subgroup; pCR, pathological complete response; PD-L1, programmed death ligand 1; SP142 VENTANA® PD-L1 SP142 assay. Database lock: March 20, 2024.

Figure S6. Proportion of patients with RCB 0/I in the nivolumab plus neoadjuvant chemotherapy and placebo plus neoadjuvant chemotherapy arms by PD-L1 status as determined by the SP142 (IC% < 1% and ≥ 1%) and 28-8 CPS (cutoffs 1–20) assays. Data are presented as percentages with error bars showing the 95% CI around the observed proportion of patients in the treatment arm. The CIs for each treatment arm were calculated using the Clopper–Pearson method and CIs for differences between treatment arms were calculated using the Newcombe method without continuity correction. The number of patients with pCR and the total number of patients in each subgroup is shown under each bar as n/N.

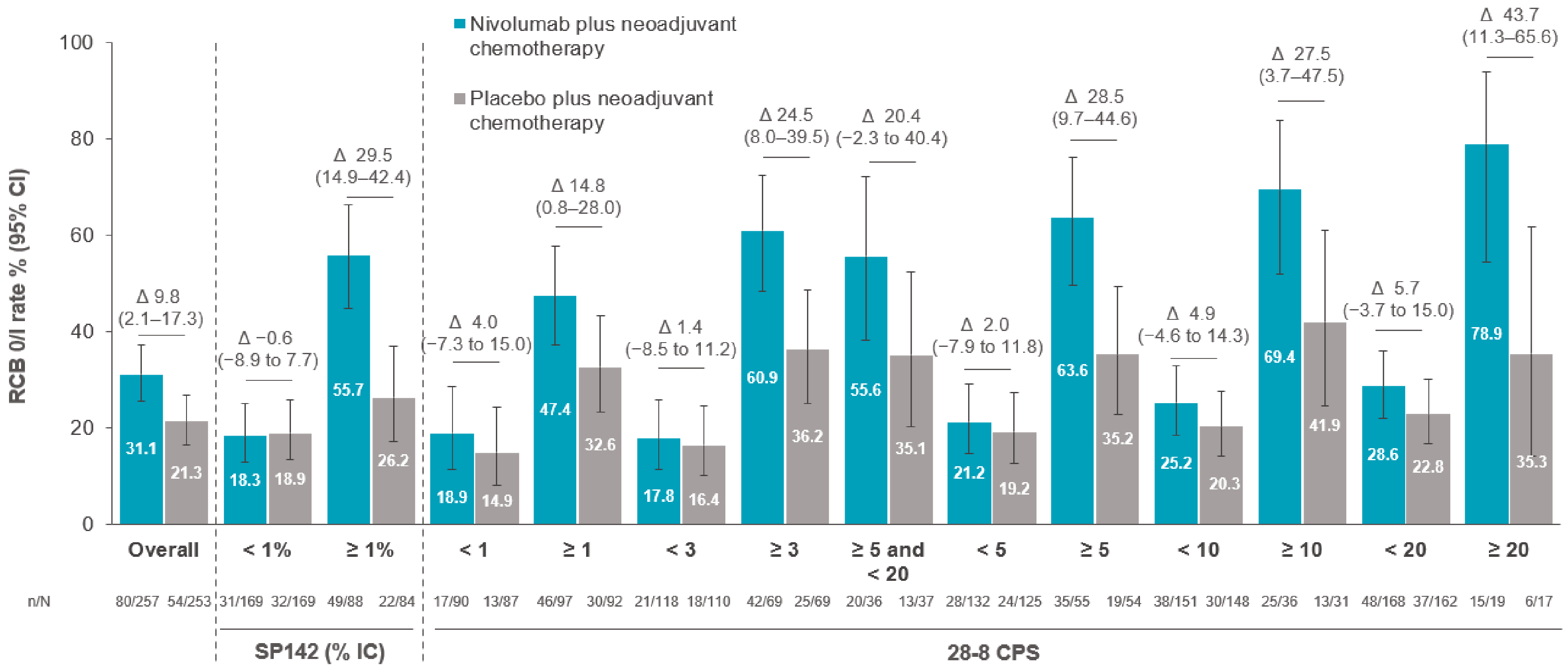

Data are presented as percentages with error bars showing the 95% CI around the observed proportion of patients in the treatment arm. The CIs for each treatment arm were calculated using the Clopper–Pearson method and CIs for differences between treatment arms were calculated using the Newcombe method without continuity correction. The number of patients with RCB 0/I and the total number of patients in each subgroup is shown below each bar (n/N). 28-8 CPS, Dako 28-8 assay using the CPS algorithm; CI, confidence interval; CPS, combined positive score; IC immune cell; n, number of patients with RCB 0/I; N, number of patients in the subgroup;PD-L1, programmed death ligand 1; RCB, residual cancer burden; SP142 VENTANA® PD-L1 SP142 assay. Database lock: March 20, 2024.

Figure S7. Prevalence of baseline PD-L1 expression in the nivolumab plus neoadjuvant chemotherapy and placebo plus neoadjuvant chemotherapy arms by SP142 IC% and sTIL at various cutoffs.

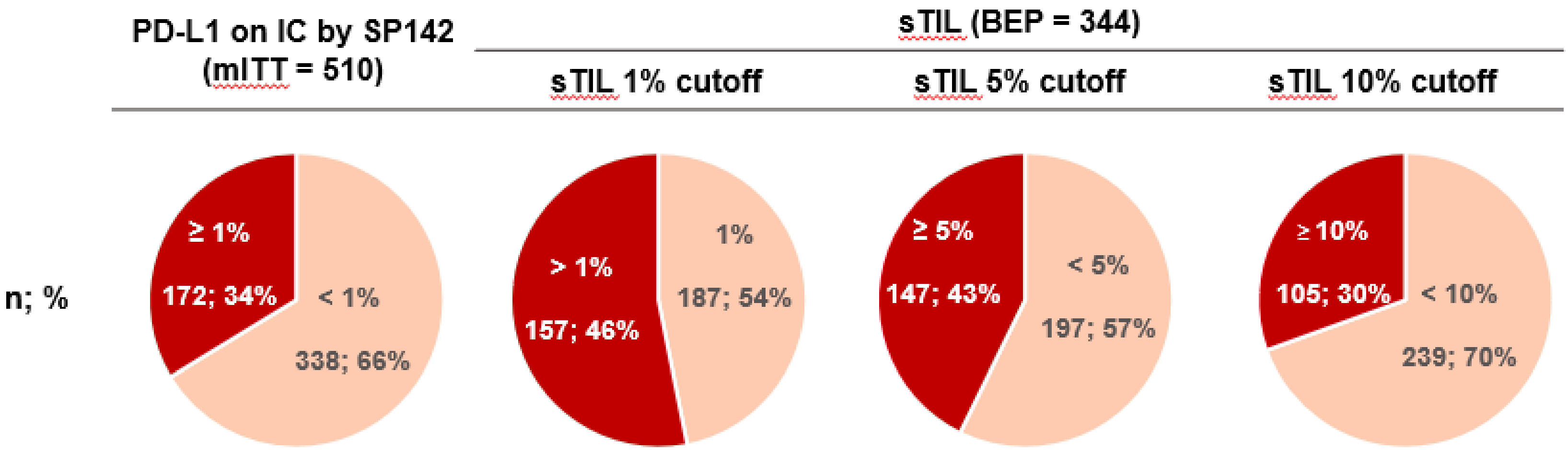

Exploratory biomarker/analysis. N = 344. BEP, biomarker-evaluable population; IC, immune cell; mITT, modified intent-to-treat; n, number of patients with PD-L1 expression; PD-L1, programmed death ligand 1; SD, standard deviation; SP142, Ventana PD-L1 SP142 assay; sTIL, stromal tumor-infiltrating lymphocyte. Database lock: March 20, 2024.

Figure S8. Interaction between PD-L1 and sTIL levels on pCR rates in the nivolumab plus neoadjuvant chemotherapy and placebo plus neoadjuvant chemotherapy arms as determined by the SP142 (% IC) and 28-8 CPS assays and percentage of sTIL at sTIL cutoffs of 1% (A) and 5% (B).

A

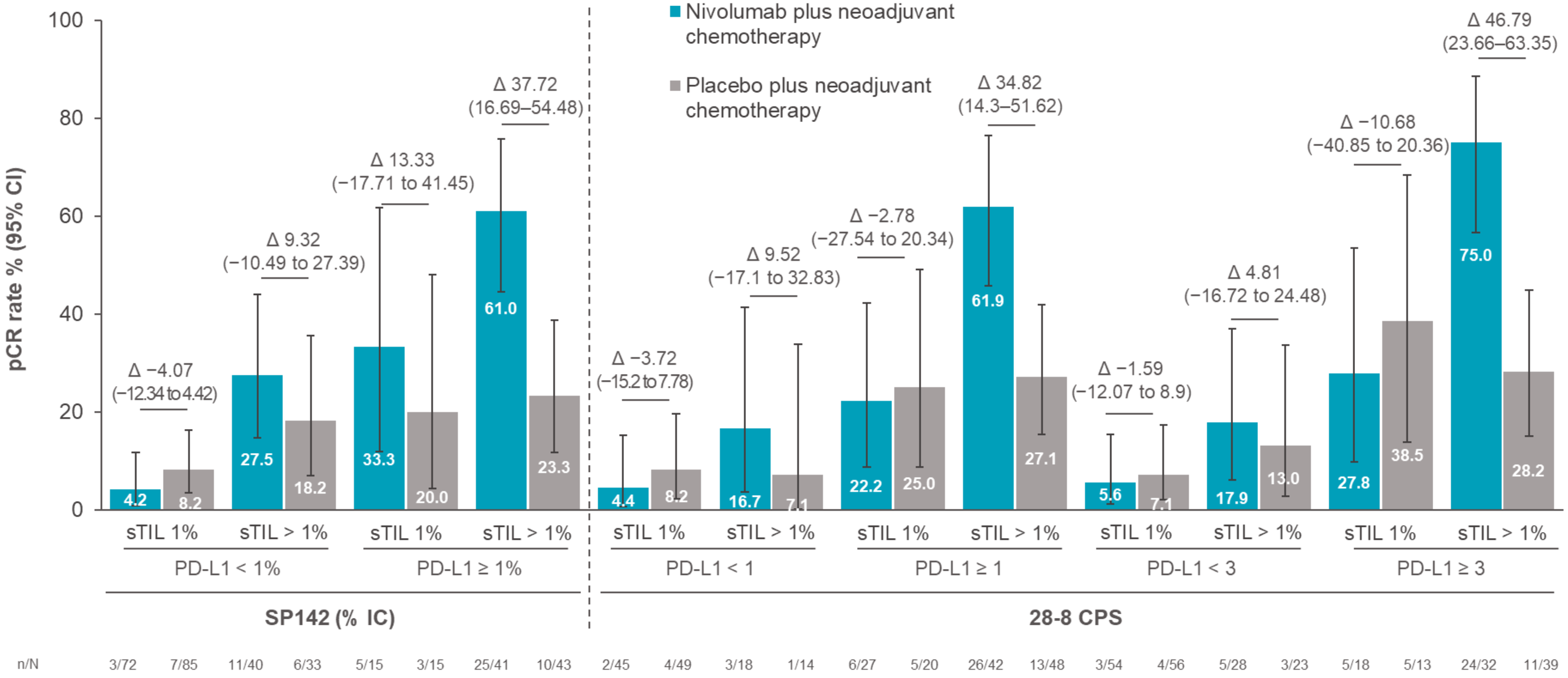

B

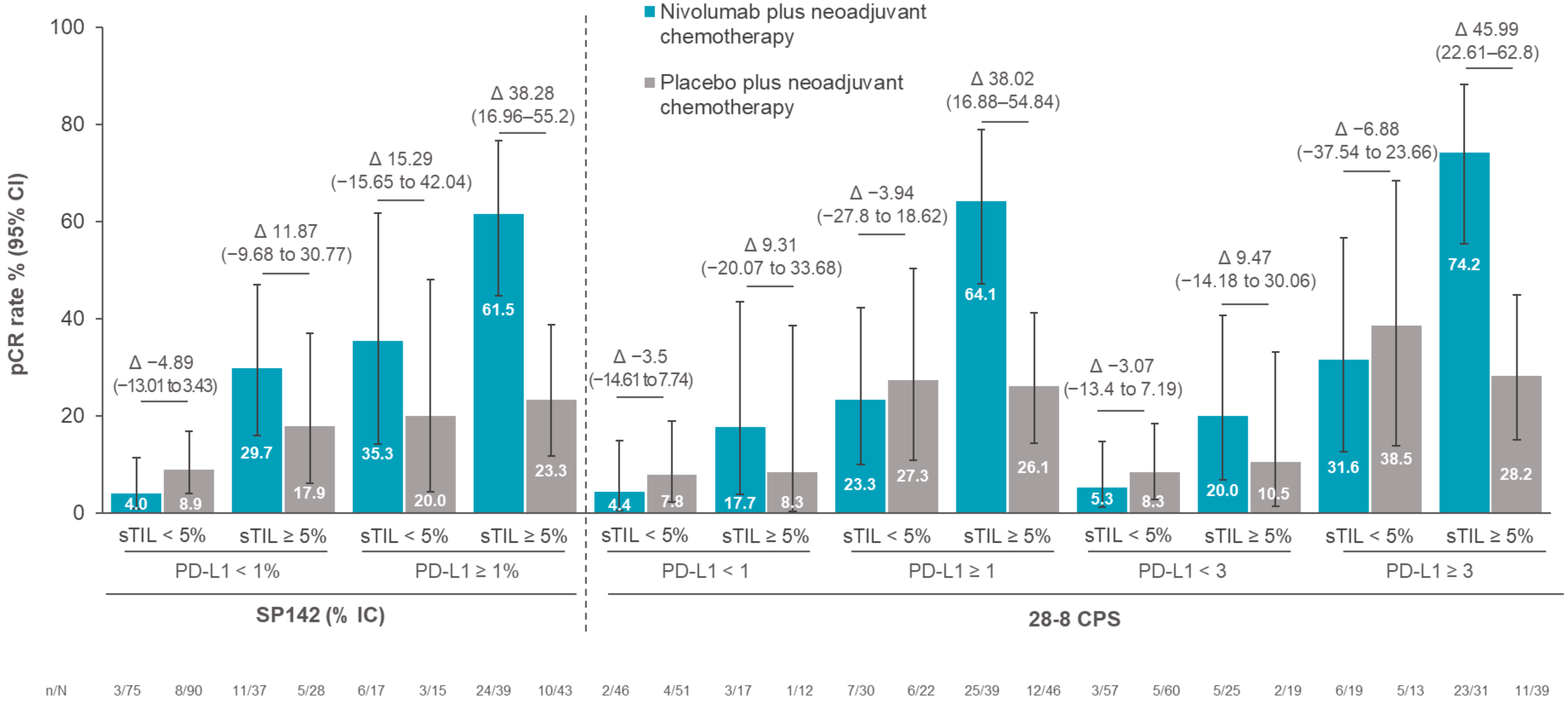

This analysis used PD-L1 on ICs per the clinical database. Data are presented as percentages with error bars showing the 95% CI around the observed proportion of patients in the treatment arm. The CIs for each treatment arm were calculated using the Clopper–Pearson method and CIs for differences between treatment arms were calculated using the Newcombe method without continuity correction. The number of patients with pCR and the total number of patients in each subgroup is shown below each bar (n/N). 28-8 CPS, Dako 28-8 assay using the CPS algorithm; CI, confidence interval; CPS, combined positive score; IC, immune cell; n, number of patients with pCR; N, number of patients in the subgroup; pCR, pathological complete response; PD-L1, programmed death ligand 1; SP142, Ventana PD-L1 SP142 assay. Database lock: March 20, 2024.

Figure S9. Proportion of patients with pCR and RCB 0/I in the nivolumab plus neoadjuvant chemotherapy and placebo plus neoadjuvant chemotherapy groups by tumor ER expression (< 10% and ≥ 10%; ≤ 50% and > 50%; ≤ 80% and > 80%).

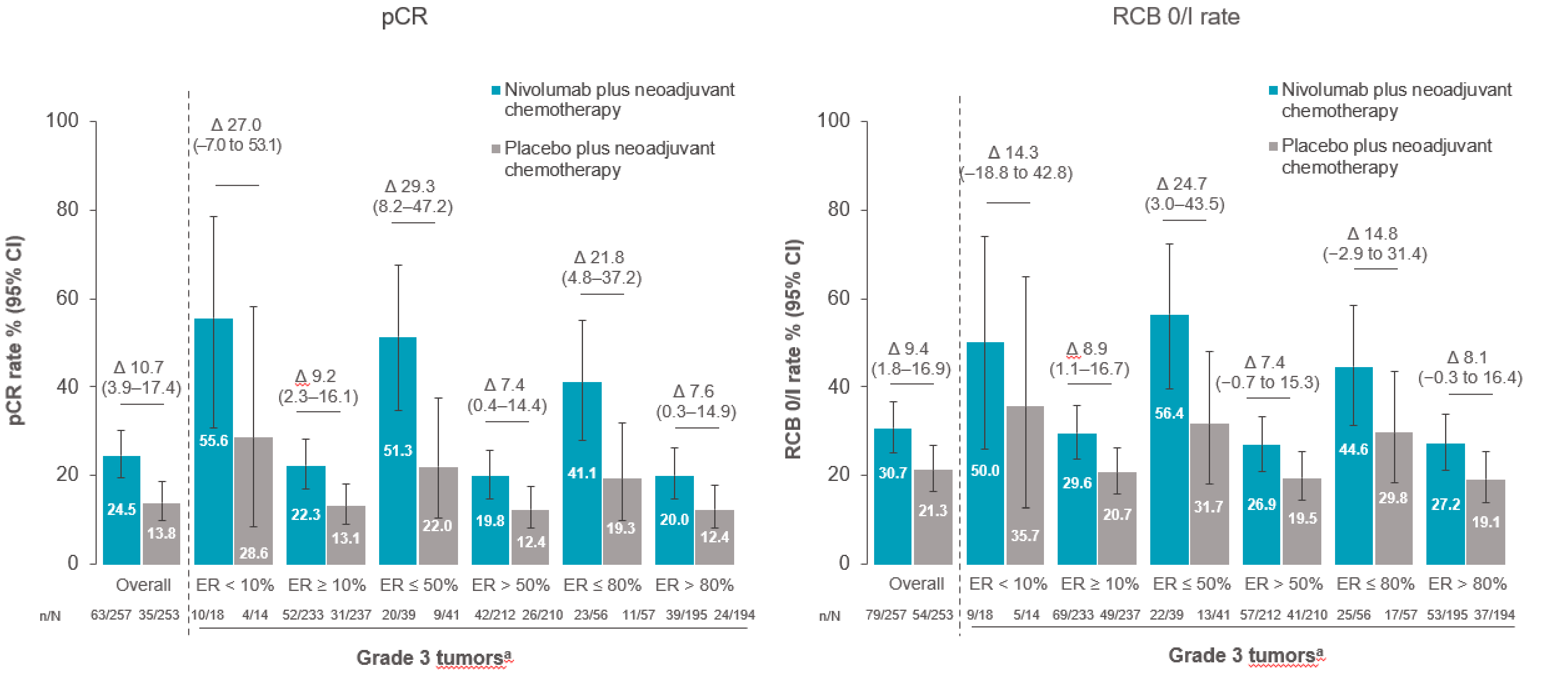

[a]Tumor grade as per the eCRF. Data are presented as percentages with error bars showing the 95% CI around the observed proportion of patients in the treatment arm. The CIs for each treatment arm were calculated using the Clopper–Pearson method and CIs for differences between treatment arms were calculated using the Newcombe method without continuity correction. The number of patients with pCR or RCB 0/I and the total number of patients in each subgroup is shown under each bar as n/N.CI, confidence interval; eCRF, electronic case report form; ER, estrogen receptor; n, number of patients with pCR or RCB 0/I; N, number of patients in the subgroup; pCR, pathological complete response; RCB, residual cancer burden. ER >50%, ER >80% and PR ≥10% were exploratory cutoffs.

Figure S10. Proportion of patients with pCR and RCB 0/I in the nivolumab plus neoadjuvant chemotherapy and placebo plus neoadjuvant chemotherapy groups by tumor PR expression (< 10% and ≥ 10%; ≤ 50% and > 50%).

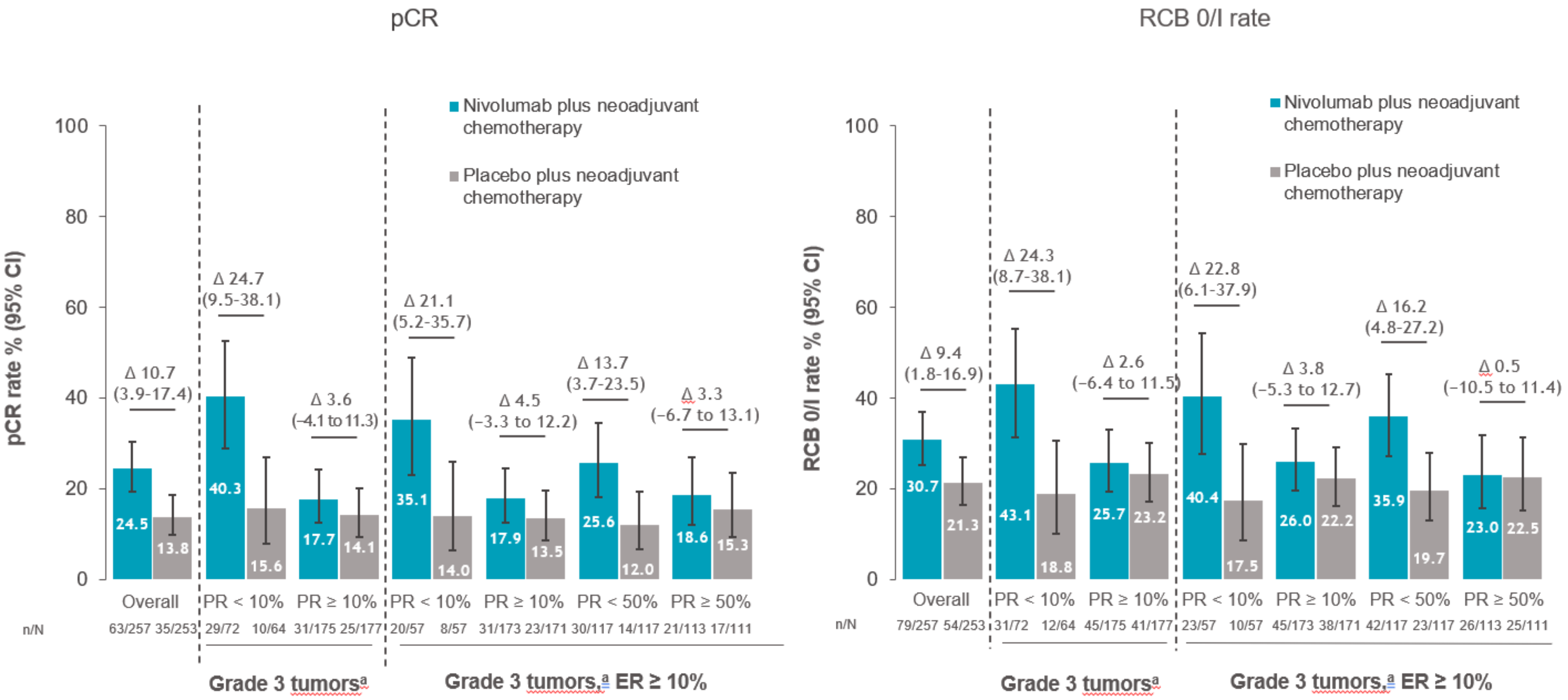

[a]Tumor grade as per the eCRF. Data are presented as percentages with error bars showing the 95% CI around the observed proportion of patients in the treatment arm. The CIs for each treatment arm were calculated using the Clopper–Pearson method and CIs for differences between treatment arms were calculated using the Newcombe method without continuity correction. The number of patients with pCR or RCB 0/I and the total number of patients in each subgroup is shown under each bar as n/N.CI, confidence interval; eCRF, electronic case report form; ER, estrogen receptor; n, number of patients with pCR or RCB 0/I; N, number of patients in the subgroup; pCR, pathological complete response; PR, progesterone receptor RCB, residual cancer burden. ER >50% ER >80% and PR ≥10% were exploratory cutoffs.

Figure S11. Proportion of patients with pCR and RCB 0/I in the nivolumab plus neoadjuvant chemotherapy and placebo plus neoadjuvant chemotherapy groups by Ki67 index (< 20% and ≥ 20%).

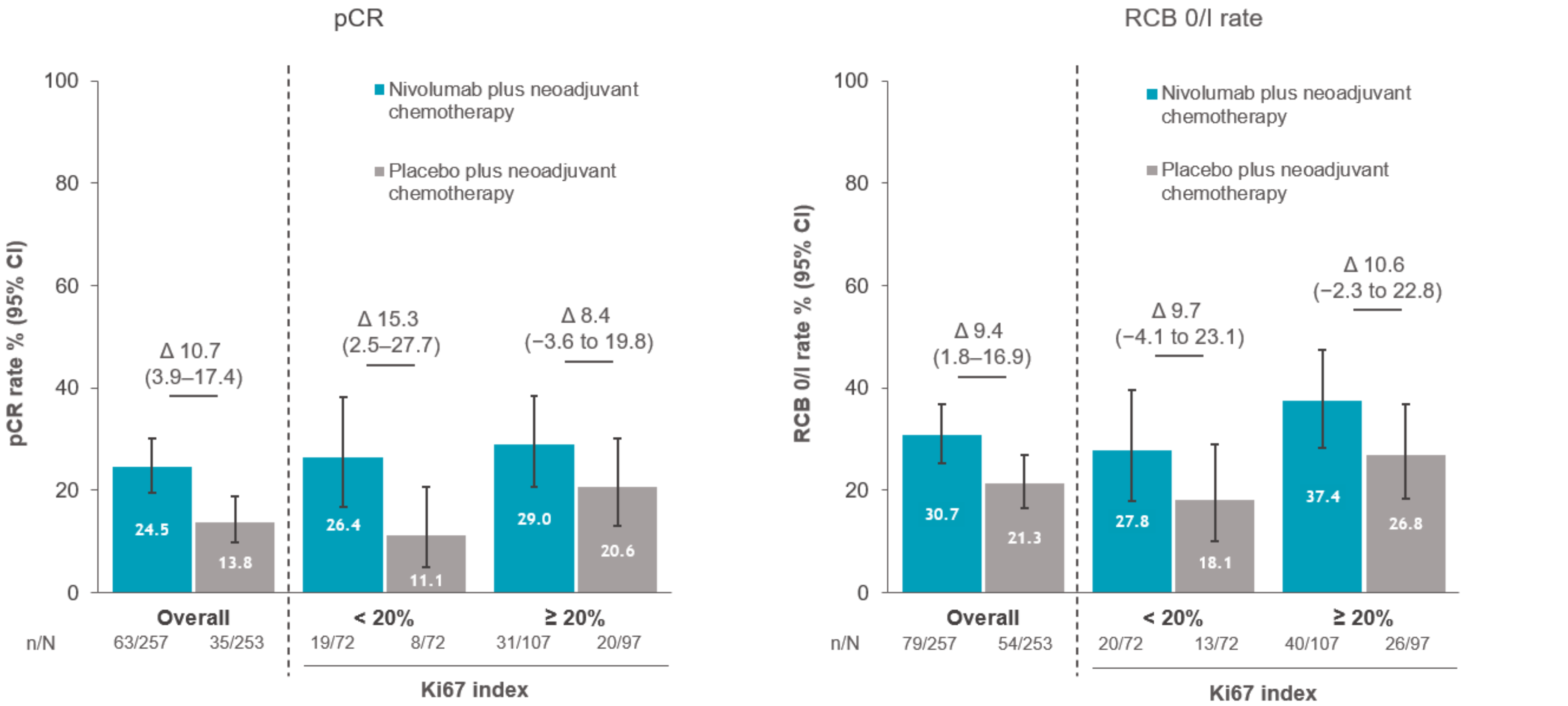

Data are presented as percentages with error bars showing the 95% CI around the observed proportion of patients in the treatment arm. The CIs for each treatment arm were calculated using the Clopper–Pearson method and CIs for differences between treatment arms were calculated using the Newcombe method without continuity correction. The number of patients with pCR or RCB 0/I and the total number of patients in each subgroup is shown under each bar as n/N. CI, confidence interval; n, number of patients with pCR or RCB 0/I; N, number of patients in the subgroup; pCR, pathological complete response; RCB, residual cancer burden.

Figure S12. Multivariable analysis of pCR in the nivolumab plus neoadjuvant chemotherapy and placebo plus neoadjuvant chemotherapy arms by biomarkers: (A) PD-L1 on ICs ≥1%with 5% sTIL cutoff, (B) PD-L1 CPS ≥3 with 5% sTIL cutoff, (C) PD-L1 on ICs ≥1% with 1% sTIL cutoff, (D) PD-L1 CPS ≥3 with 1% sTIL cutoff, and other baseline disease characteristics

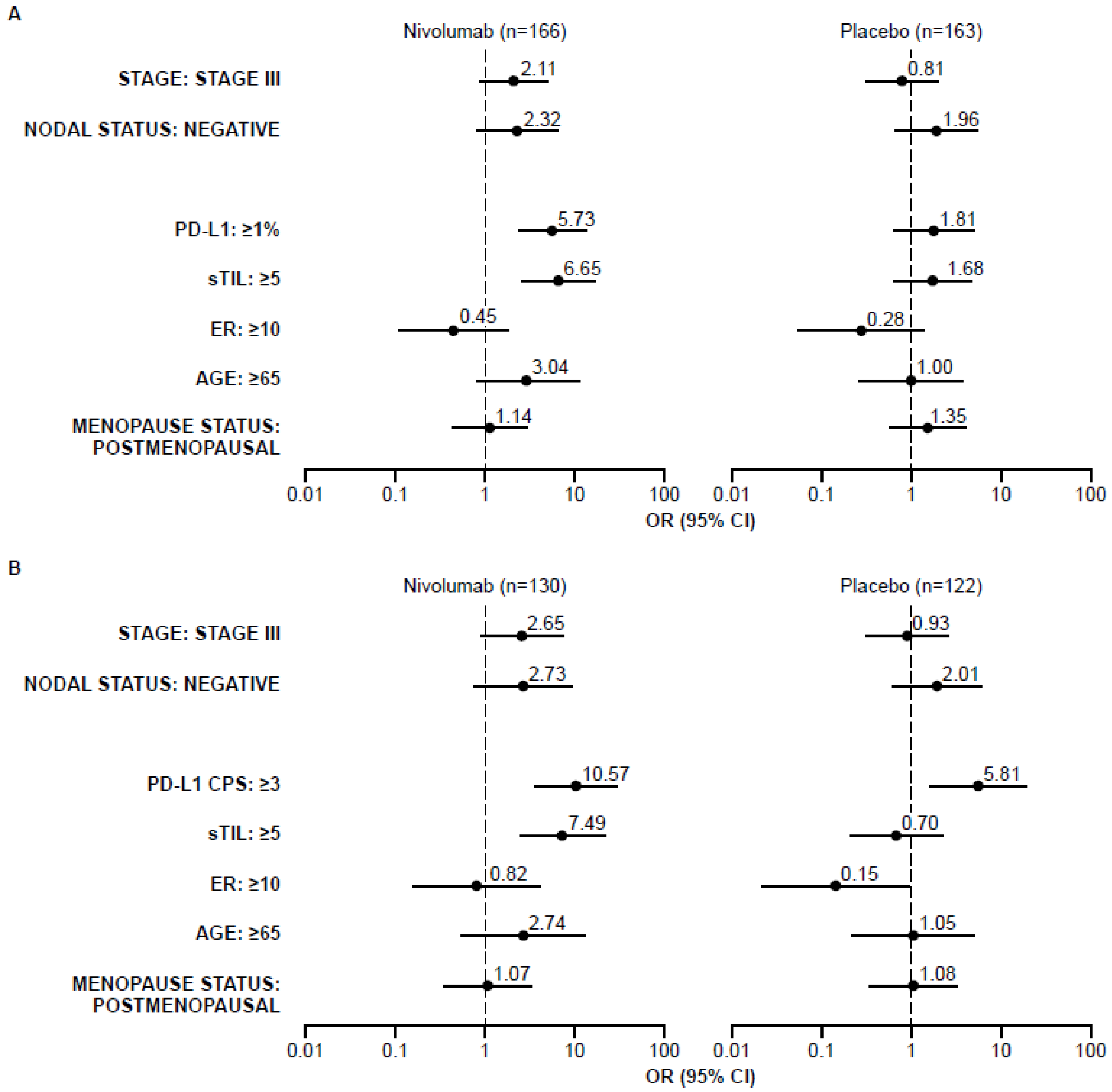

C

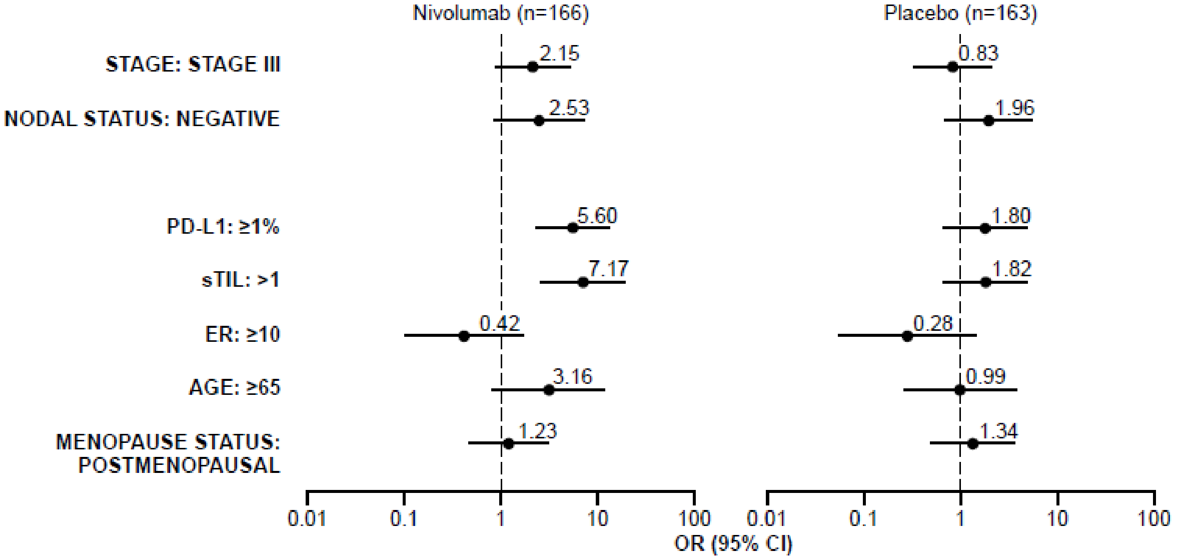

D

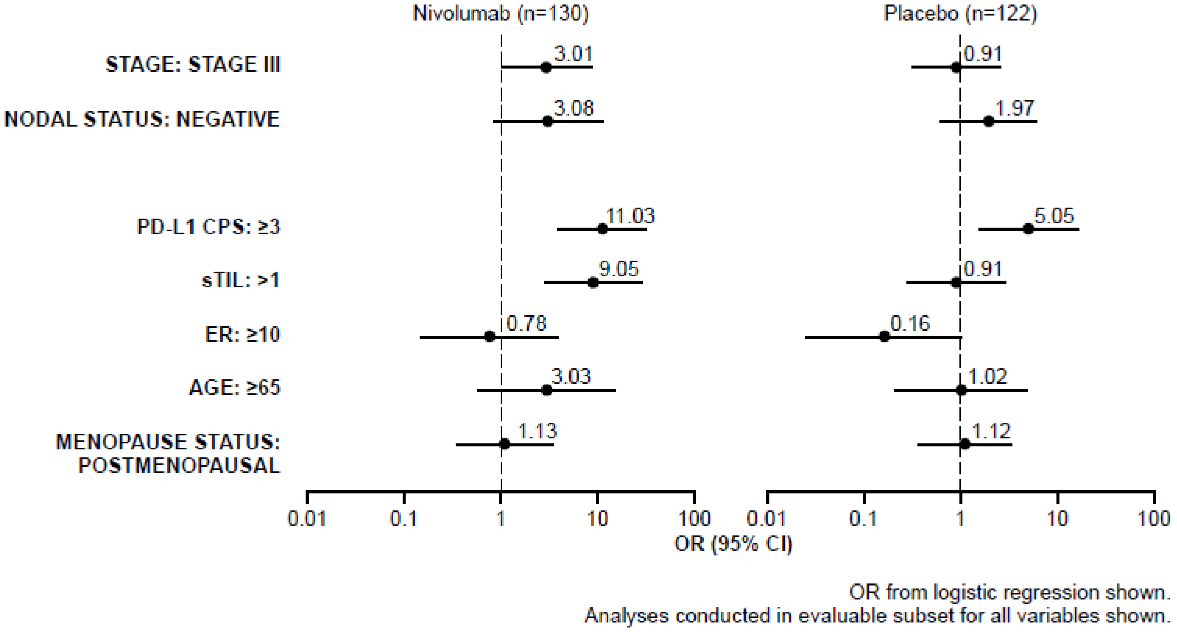

Data are presented as percentages with error bars showing the 95% CI around the observed proportion of patients in the treatment arm. This analysis uses PD-L1 on ICs per the clinical database. Data are presented as ORs with error bars showing the 95% CI (from logistic regression) around the OR. Biomarkers were analyzed as categorical variables. CI, confidence interval; CPS, combined positive score; ER, estrogen receptor; IC, immune cell; OR, odds ratio; pCR, pathological complete response; PD-L1, programmed death ligand 1; sTIL, stromal tumor-infiltrating lymphocyte. Database lock: March 20, 2024.

Figure S13. Multivariable analysis of pCR in the nivolumab plus neoadjuvant chemotherapy and placebo plus neoadjuvant chemotherapy arms by biomarkers: (A) PD-L1 on IC as a continuous variable and (B) PD-L1 CPS as a continuous variable, and other baseline disease characteristics.

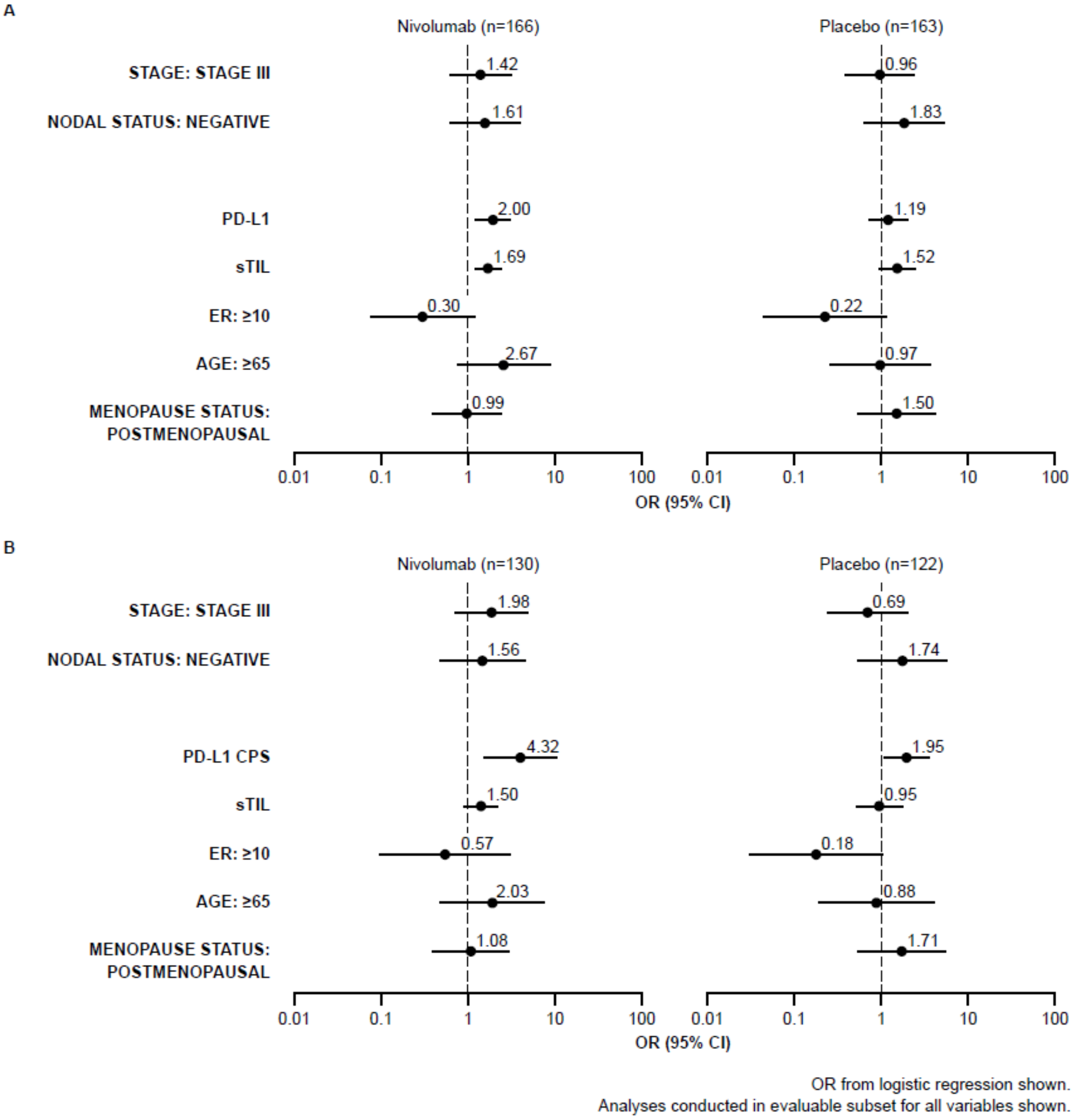

This analysis uses PD-L1 on ICs per the clinical database. Analyses conducted in evaluable subset for all variables shown. Data are presented as ORs with error bars showing the 95% CI (from logistic regression) around the OR. Biomarkers were analyzed as continuous variables. CI, confidence interval; CPS, combined positive score; ER, estrogen receptor; IC, immune cell; OR, odds ratio; pCR, pathological complete response; PD-L1, programmed death ligand 1; sTIL, stromal tumor-infiltrating lymphocyte. Database lock: March 20, 2024.
