# Supplementary material for: Neoadjuvant nivolumab and chemotherapy in early estrogen receptor-positive breast cancer: a randomized phase 3 trial
Source: Nat Med. 2025 Jan 21;31(2):433–41. doi: 10.1038/s41591-024-03414-8 (PMC11835735; doi:10.1038/s41591-024-03414-8)
Supplement: Supplementary file 2 — Reporting Summary [file 41591_2024_3414_MOESM2_ESM.pdf]

nature portfolio

Corresponding author(s): Sherene Loi

Last updated by author(s): Oct 17, 2024

Reporting Summary

Nature Portfolio wishes to improve the reproducibility of the work that we publish. This form provides structure for consistency and transparency in reporting. For further information on Nature Portfolio policies, see our Editorial Policies and the Editorial Policy Checklist.

Statistics

For all statistical analyses, confirm that the following items are present in the figure legend, table legend, main text, or Methods section.

n/a	Confirmed	
☐	☒	The exact sample size (*n*) for each experimental group/condition, given as a discrete number and unit of measurement
☒	☐	A statement on whether measurements were taken from distinct samples or whether the same sample was measured repeatedly
☐	☒	The statistical test(s) used AND whether they are one- or two-sided<br>*Only common tests should be described solely by name; describe more complex techniques in the Methods section.*
☐	☒	A description of all covariates tested
☐	☒	A description of any assumptions or corrections, such as tests of normality and adjustment for multiple comparisons
☐	☒	A full description of the statistical parameters including central tendency (e.g. means) or other basic estimates (e.g. regression coefficient) AND variation (e.g. standard deviation) or associated estimates of uncertainty (e.g. confidence intervals)
☐	☒	For null hypothesis testing, the test statistic (e.g. *F*, *t*, *r*) with confidence intervals, effect sizes, degrees of freedom and *P* value noted<br>*Give P values as exact values whenever suitable.*
☒	☐	For Bayesian analysis, information on the choice of priors and Markov chain Monte Carlo settings
☒	☐	For hierarchical and complex designs, identification of the appropriate level for tests and full reporting of outcomes
☒	☐	Estimates of effect sizes (e.g. Cohen's *d*, Pearson's *r*), indicating how they were calculated

Our web collection on statistics for biologists contains articles on many of the points above.

Software and code

Policy information about availability of computer code

Data collection	No software was used
Data analysis	No software was used

For manuscripts utilizing custom algorithms or software that are central to the research but not yet described in published literature, software must be made available to editors and reviewers. We strongly encourage code deposition in a community repository (e.g. GitHub). See the Nature Portfolio guidelines for submitting code & software for further information.

Data

Policy information about availability of data

All manuscripts must include a data availability statement. This statement should provide the following information, where applicable:

- Accession codes, unique identifiers, or web links for publicly available datasets
- A description of any restrictions on data availability
- For clinical datasets or third party data, please ensure that the statement adheres to our policy

Bristol Myers Squibb will honor legitimate requests for clinical trial data from qualified researchers with a clearly defined scientific objective. Bristol Myers Squibb will consider data sharing requests for Phase II-IV interventional clinical trials that completed on or after January 1, 2008. In addition, primary results from these trials must have been published in peer-reviewed journals and the medicines or indications approved in the U.S., EU, and other designated markets. Sharing is also

subject to protection of patient privacy and respect for the patient's informed consent. Data considered for sharing may include non-identifiable patient-level and study-level clinical trial data, full clinical study reports and protocols.

Research involving human participants, their data, or biological material

Policy information about studies with human participants or human data. See also policy information about sex, gender (identity/presentation), and sexual orientation and race, ethnicity and racism.

Reporting on sex and gender	The sex of patients enrolled in the trial was self-reported and data for gender were not collected. The eligible patient population was predominantly female, and the trial enrolled a single male patient; no analyses by sex or disaggregated data were presented because doing so would result in presentation of potentially identifying information.
Reporting on race, ethnicity, or other socially relevant groupings	Provided in Table 1
Population characteristics	Provided in Table 1
Recruitment	From November 20, 2019 through April 07, 2022, 830 patients were screened at 221 clinical sites in 31 countries. Of the 830 patients screened, 521 were randomized. Eligible patients had newly diagnosed ER+/HER2− breast cancer, with a confirmed primary tumor and node categories of tumors sized 2–5 cm and cN1–cN2 or cT3–cT4 and cN0–cN2; grade 3 disease or grade 2 disease with ER expression of 1 to ≤10%; adequate organ function; tissue available for biomarker assessment; and Eastern Cooperative Oncology Group performance status 0–1. Patients were eligible irrespective of PD-L1 status. Multifocal tumors (two or more foci of cancer within the same breast quadrant) were permitted if the largest lesion was at least 2 cm and designated as the target lesion. Patients were excluded if they had multicentric breast cancer, a history of ipsilateral invasive breast cancer, evidence of metastatic disease, had received any prior treatment for the currently diagnosed breast cancer, or had received prior immunotherapy.
Ethics oversight	This trial was developed and overseen by an academic steering committee and employees of the sponsor (Bristol Myers Squibb). An external, independent data monitoring committee provided oversight of safety and efficacy considerations during the study. The trial protocol and amendments were approved by the appropriate ethics body at each participating site. All patients provided written informed consent. All authors confirm that the trial was conducted with respect to the standards of Good Clinical Practice.

Note that full information on the approval of the study protocol must also be provided in the manuscript.

Field-specific reporting

Please select the one below that is the best fit for your research. If you are not sure, read the appropriate sections before making your selection.

☒ Life sciences ☐ Behavioural & social sciences ☐ Ecological, evolutionary & environmental sciences

For a reference copy of the document with all sections, see nature.com/documents/nr-reporting-summary-flat.pdf

Life sciences study design

All studies must disclose on these points even when the disclosure is negative.

Sample size	A sample size of 521 patients in the intent-to-treat population would yield 87% power (two-sided alpha 0.05) to detect a difference of 10% in pCR rates between treatment arms, assuming a 12% pCR rate in the control arm. Due to the sponsor's decision to close all sites in Russia, 11 patients were excluded due to insufficient follow-up for pCR assessment, with a small impact on the study power (86%).
Data exclusions	Due to the sponsor's decision to close all sites in Russia, 11 patients were excluded due to insufficient follow-up for pCR assessment.
Replication	The study started enrolling patients from 31 countries in November 2019 and completed in December 2023. Each participating patient was followed from the time of enrollment until study discontinuation or death. As such, replication of the study was not possible.
Randomization	Randomization was stratified per interactive response technology by the proportion of PD-L1-expressing immune cells (percentage of immune cells by VENTANA® PD-L1 SP142 immunohistochemistry, cut-off at 1%), tumor grade (2 or 3), pathologically confirmed axillary nodal status (positive on pathological review or negative on radiographic and/or pathologic review) and anthracycline dosing frequency (every 3 weeks or every 2 weeks).
Blinding	Patients, those involved with their management and those collecting and analyzing the data were blinded; however, as per the protocol amendment, the study was unblinded in the adjuvant phase following the approval of adjuvant abemaciclib for high-risk primary ER+/HER2. The combination of abemaciclib with nivolumab was expected to result in a high rate of withdrawals due to safety concerns around combining a CDK4/6 inhibitor with an anti–PD-1 agent. Per the protocol amendment, in the adjuvant phase, patients received nivolumab 480 mg with investigator's choice of ET (tamoxifen, letrozole, anastrozole, or exemestane, with or without ovarian function suppression) for up to seven cycles.

Reporting for specific materials, systems and methods

We require information from authors about some types of materials, experimental systems and methods used in many studies. Here, indicate whether each material, system or method listed is relevant to your study. If you are not sure if a list item applies to your research, read the appropriate section before selecting a response.

Materials & experimental systems

n/a	Involved in the study
☐	☒ Antibodies
☒	☐ Eukaryotic cell lines
☒	☐ Palaeontology and archaeology
☒	☐ Animals and other organisms
☐	☒ Clinical data
☒	☐ Dual use research of concern
☒	☐ Plants

Methods

n/a	Involved in the study
☒	☐ ChIP-seq
☒	☐ Flow cytometry
☒	☐ MRI-based neuroimaging

Antibodies

Antibodies used	PD-L1 was evaluated by qualitative immunohistochemistry on immune cells with the VENTANA® SP142 assay (Roche Diagnostics) and PD-L1 combined positive score with the 28-8 pharmDx assay (Agilent).
Validation	*Describe the validation of each primary antibody for the species and application, noting any validation statements on the manufacturer's website, relevant citations, antibody profiles in online databases, or data provided in the manuscript.*

Clinical data

Policy information about clinical studies

All manuscripts should comply with the ICMJE guidelines for publication of clinical research and a completed CONSORT checklist must be included with all submissions.

Clinical trial registration	NCT04109066
Study protocol	The protocol is available as a supplement to this publication.
Data collection	Clinical data was collected at participating sites (clinical research institutes and hospitals) from November 20, 2019 to April 07, 2022. The study was conducted at 221 sites in 31 countries (Argentina, Australia, Austria, Belgium, Brazil, Canada, Chile, China, Colombia, Czech Republic, Denmark, Finland, France, Germany, Hong Kong, Ireland, Italy, Republic of Korea, Mexico, Netherlands, Poland, Portugal, Romania, Russian Federation [BMS terminated all activities in Russia on June 2022], Singapore, Spain, Switzerland, Taiwan, Turkey, United Kingdom, and USA)
Outcomes	The primary endpoint was pCR (ypT0/is, ypN0) in the modified intent-to-treat population. Following the decision to discontinue enrollment in the study in April 2022 due to the changing treatment landscape, event-free survival was changed from a co-primary endpoint to an exploratory endpoint, as the total number of enrolled patients was too low to provide sufficient power for comparison. Consequently, the follow-up was reduced to 1 year post-surgery for all patients, and the adjuvant phase became open-label. Secondary endpoints included pCR in the PD-L1+ population, and RCB 0/I rate in the modified intent-to-treat and PD-L1+ populations. Pathological response was assessed, and RCB score calculated by local pathologists. The RCB score combined tumor size, tumor cellularity, and nodal involvement into a single continuous score that was grouped into four classes, namely, RCB score of 0 (i.e. pathologic complete response), and I, II, and III. Safety and tolerability were assessed during the neoadjuvant and adjuvant phases in all patients who received at least one dose of study drug. Prespecified exploratory endpoints included association of efficacy outcomes by biomarker status based on PD-L1 combined positive score , stromal tumor-infiltrating lymphocytes (sTILs), levels of estrogen/progesterone receptors, and Ki67 index.

Plants

Seed stocks	*Report on the source of all seed stocks or other plant material used. If applicable, state the seed stock centre and catalogue number. If plant specimens were collected from the field, describe the collection location, date and sampling procedures.*
Novel plant genotypes	*Describe the methods by which all novel plant genotypes were produced. This includes those generated by transgenic approaches, gene editing, chemical/radiation-based mutagenesis and hybridization. For transgenic lines, describe the transformation method, the number of independent lines analyzed and the generation upon which experiments were performed. For gene-edited lines, describe the editor used, the endogenous sequence targeted for editing, the targeting guide RNA sequence (if applicable) and how the editor was applied.*
Authentication	*Describe any authentication procedures for each seed stock used or novel genotype generated. Describe any experiments used to assess the effect of a mutation and, where applicable, how potential secondary effects (e.g. second site T-DNA insertions, mosiacism, off-target gene editing) were examined.*
